# Photocatalytic Difluoromethylation Reactions of Aromatic Compounds and Aliphatic Multiple C–C Bonds

**DOI:** 10.3390/molecules24244483

**Published:** 2019-12-06

**Authors:** Sebastián Barata-Vallejo, Al Postigo

**Affiliations:** Universidad de Buenos Aires, Facultad de Farmacia y Bioquímica, Departamento de Química Orgánica, Junín 954, Buenos Aires CP1113, Argentina; sbaratavallejo@ffyb.uba.ar

**Keywords:** difluoromethylation, photocatalytic difluoromethylation, visible light, difluoromethyl-substituted (hetero)arenes, difluoromethylation of olefins

## Abstract

Among the realm of visible light photocatalytic transformations, late-stage difluoromethylation reactions (introduction of difluoromethyl groups in the last stages of synthetic protocols) have played relevant roles as the CF_2_X group substitutions exert positive impacts on the physical properties of organic compounds including solubility, metabolic stability, and lipophilicity, which are tenets of considerable importance in pharmaceutical, agrochemical, and materials science. Visible-light-photocatalyzed difluoromethylation reactions are shown to be accomplished on (hetero)aromatic and carbon–carbon unsaturated aliphatic substrates under mild and environmentally benign conditions.

## 1. Introduction

The relevance of organofluorine compounds spans areas of considerable importance such as pharmaceutical, agrochemical, and materials science, as the introduction of fluoro-substituents into these types of molecules has a striking positive impact on their physical properties including metabolic stability, solubility, and lipophilicity. The fluorine atom is widely considered to be a valuable heteroatomic substitute for H. Fluorinated groups such as trifluoromethyl, trifluoromethoxy, trifluoroethyl, and perfluoroalkyl are common fluorinated scaffolds known to contribute with relevant changes in substrates with known biological activity.

**Figure 1:**
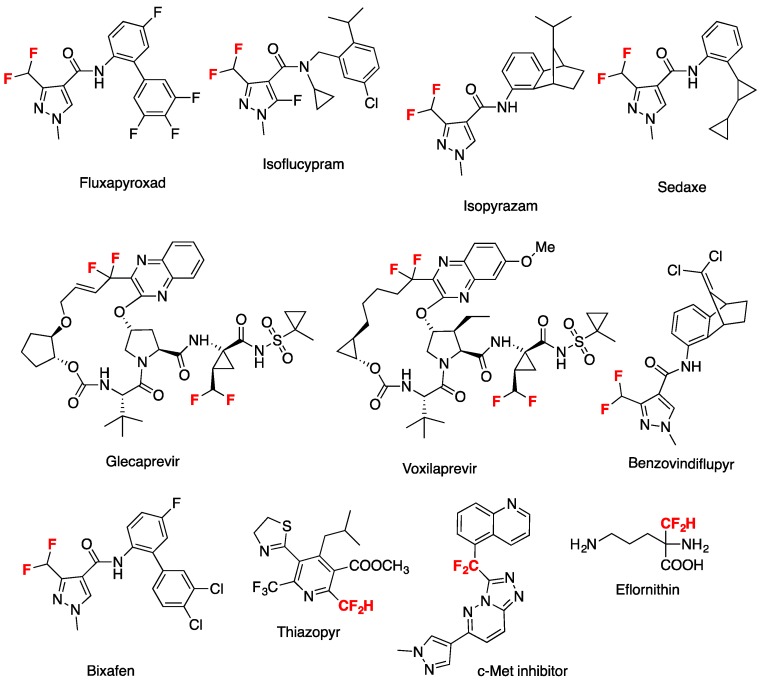
Federal Drug Administration-approved drugs that contain the difluoromethyl group.

Among other fluoroalkyl groups, the difluoromethyl group has garnered particular attention in medicinal chemistry due to the fact that the CF_2_H moiety is isosteric and isopolar to the –OH and –SH groups, acting as a lipophilic hydrogen bond donor. This simultaneously harnesses the electronegativity of the fluorine atoms to emulate the oxygen (or sulfur) lone electron pairs, while rendering the methane proton acidic and a competent hydrogen bond donor [1]. The presence of the CF_2_ group in a compound can also induce conformational changes and dipole moments, along with increasing the acidity of neighboring groups, thus modulating the pKa of proximal heteroatoms in heterocycles. Several FDA-approved drugs contain the CF_2_ group (Figure 1), and 74 drugs the CF_3_ one [2]. On account of the former, considerable research efforts have been geared toward the efficient introduction of difluoromethyl groups into organic compounds. However, when compared to the well-established trifluoromethylations, methods for the corresponding difluoromethylation reactions remain scarce and challenging.

**Figure 2:**
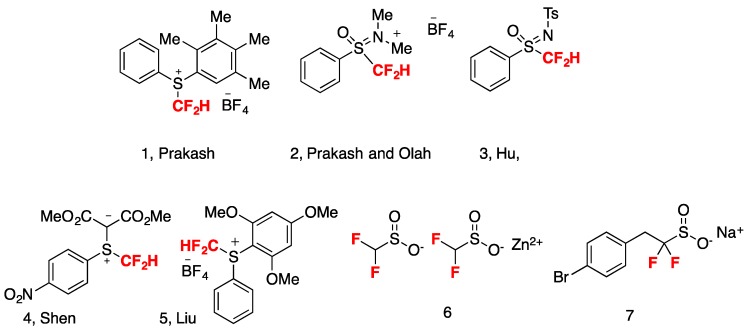
Electrophilic difluoromethylating reagents and structures of zinc difluoromethylsulfinate **6** and sodium 2-(4-bromophenyl)-1,1-difluoroethanesulfinate **7**.

Different approaches for the syntheses of compounds containing the difluoromethyl moiety have been reported including the deoxofluorination of aldehydes with SF_4_, DAST (*N,N*-diethylaminosulfur trifluoride) and its derivatives [3,4] as well as nucleophilic, electrophilic (**1**–**5**, Figure 2), and radical difluoromethylations [5,6,7,8,9,10,11,12,13]. Alternatively, the difluoromethylation of heteroatom nucleophiles with difluorocarbene reagents has also been developed for the direct preparation of compounds containing the difluoromethyl group [14,15,16,17,18,19].

Other commercially available reagents such as sodium difluoromethanesulfinate NaSO_2_CF_2_H, zinc difluoromethylsulfinate **6** [20], sodium 2-(4-bromophenyl)-1,1-difluoroethanesulfinate **7** (Baran’s reagents) are now currently employed in difluoromethylation radical chemistry reactions (Figure 2).

The study of synthetic pathways to introduce the CF_2_–Y moiety (Y ≠ H, F) into organic substrates is also relevant, since compounds that contain the CF_2_–Y functionality have also found vast applications in medicinal chemistry and in other areas such as that of fungicides, insecticides, etc., which therefore deserve special attention.

A growing number of radical difluoromethylation reactions can be accomplished through the aid of visible light and the use of (organo)metallic photocatalysts [21,22] that harvest light in the form of energy to generate reactive species that enable numerous transformations toward synthetic targets needed in fields such as natural product synthesis, late-stage pharmaceutical functionalization, and polymer synthesis. Mechanistic details regarding photocatalytic organic reactions are providing new insights into the processes involved, consolidating a thorough comprehension of the scope and role of photocatalysts in organic synthetic transformations [23,24,25,26,27,28]. This new focus on photocatalysis (i.e., combining synthetic and mechanistic detailed studies of photocatalytic reactions) departs from the conventional mere synthetic approach utilized in the early applications of organic photocatalysis [23].

Among the realm of visible light photocatalytic transformations [29,30], late-stage difluoromethylation reactions (introduction of difluoromethyl groups in the last stages of synthetic protocols) have played, as stated before, relevant roles, as the CF_2_X group substitutions exert positive impacts on the physical properties of organic compounds including solubility, metabolic stability, and lipophilicity, which are tenets of considerable importance in pharmaceutical, agrochemical, and materials science when designing targets of biological importance and building blocks.

The strategy presented in this paper to achieve late-stage difluoromethylation reactions are going to be photocatalytic in nature. In 2017, there were review articles [31,32] describing the up-to-then difluoromethylation strategies to achieve late-stage CF_2_X introduction into organic molecules. More recently, Koike and Akita [33] have summarized the radical mono- and difluoromethylation reactions accomplished photochemically.

A great deal of research activity on the area has evolved since the 2017 accounts have appeared, especially involving visible-light photocatalytic methods, which justifies a comprehensive and integral treatment of the new difluoromethylation reactions that have been published since.

To aid the organic synthetic chemist and the researcher in the field of fluorination techniques, we organized the paper into different families of (hetero)aromatic and unsaturated aliphatic substrates where the late difluoromethyl group is introduced by means of activation with visible light. A thorough mechanistic interpretation also accompanies the selected examples.

## 2. Photocatalytic Difluoromethylation of (Hetero)aromatic Compounds

### 2.1. C_(Het)_-H Difluoromethylation of (Hetero)aromatic Compounds

Research into site-selective C_Ar_–H difluoromethylation of arenes has been of considerable importance. For example, the Ackermann group developed a ruthenium-catalyzed *meta*-selective C–H difluoromethylation of 2-arylpyridine derivatives [34].

**Scheme 1:**
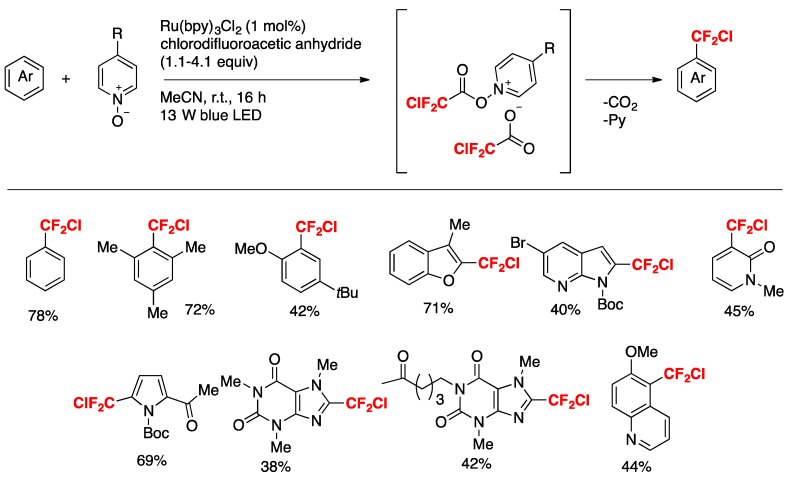
Selected examples of chlorodifluoromethylation of (hetero)arenes.

McAtee, Stephenson, and colleagues [35] have informed the chloro-difluoromethylation of (hetero)arenes. While the difluoromethyl radical CF_2_H exhibits nucleophilic behavior, the chlorodifluoromethyl CF_2_Cl radical may be characterized as an electrophilic radical. Therefore, the CF_2_Cl radical can be considered as an attractive surrogate to the difluoromethyl radical and a means to efficiently prepare electron-rich difluoromethylated (hetero)arenes and other high-value, fluorinated heterocycles. The authors in [35] employed commercially available chlorodifluoroacetic anhydride as a source of CF_2_Cl radicals and found that with benzene as the substrate, a combination of pyridine-*N*-oxide, chlorodifluoroacetic anhydride, photocatalyst Ru(bpy)_2_^2+^ (1 mol%) and blue LEDs furnished the desired chlorodifluoromethylated benzene in very good yield (78%). With the optimized reaction conditions in hand, the scope of the reaction was investigated, and relevant examples are shown in Scheme 1.

**Scheme 2:**
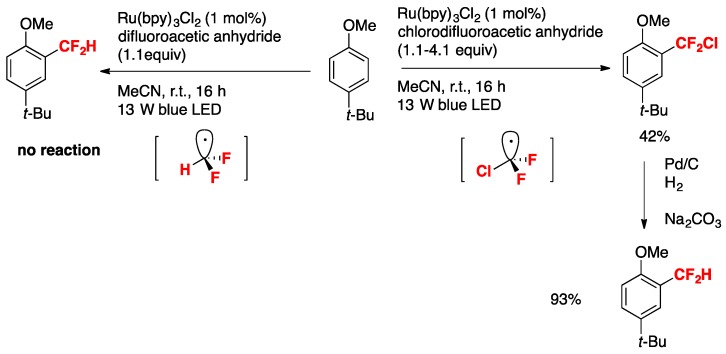
Electronic effects of difluoroalkyl radicals with *p-tert*-butylanisole.

The authors in [35] also demonstrated the importance of the electronic effects governing the substrate reactivity and the difluoromethyl radical. For an electron-rich substrate such as the *p-tert*-butylanisole, the authors in [35] studied the reactivity versus the chlorodifluoromethyl radical and the difluoromethyl radical such as is illustrated in Scheme 2.

In Scheme 2, the difluoromethyl (CF_2_H) radical generated from difluoroacetic anhydride, pyridine-*N*-oxide under Ru-photocatalysis does not react with electron-rich *p-tert*-butylanisole, whereas the chlorodifluoromethyl radical ClCF_2_ (produced from chlorodifluoroacetic anhydride, pyridine-*N*-oxide under photocatalysis) affords 42% yield of 2-chlorodifluoromethyl-4-*tert*-butylanisole, which upon hydrogenolysis renders 2-difluoromethyl-4-*tert*-butylanisole in 93% yield. As the CF_2_Cl radical is more electrophilic than the CF_2_H radical, the former will react with electron-rich positions of the aromatic nucleus (i.e., position 2-), while the latter with more nucleophilic positions. Therefore, the higher yield obtained for the *p*-*tert*-butyl-2-difluorochloromethylanisole product is expected.

**Scheme 3:**
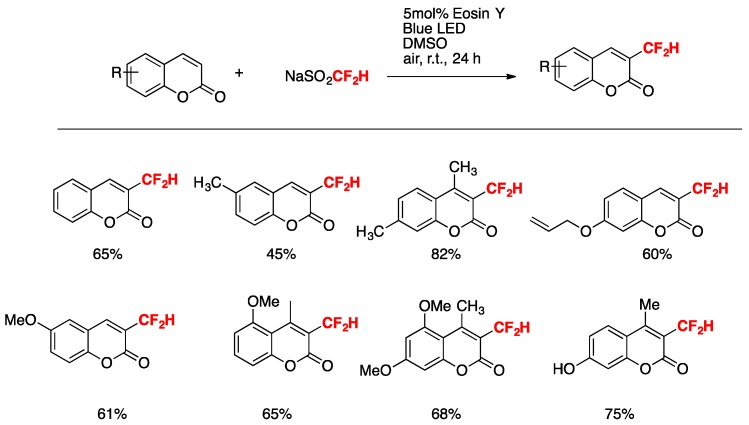
Selected examples for the difluoromethylation reaction of coumarins.

Dai, Zhang, Deng, and colleagues [36] accomplished the difluoromethylation of coumarins by the Eosin Y -blue-LED-photocatalyzed reaction in the presence of NaSO_2_CF_2_H in DMSO as the solvent under air at room temperature. The optimized reaction conditions involve 0.3 mmol of coumarin, 3 equiv. of NaSO_2_CF_2_H, and 5 mol% of photocatalyst in dimethylsulfoxide (DMSO) as the solvent, irradiated with a blue LED for 24 h at room temperature. The scope of the transformation is illustrated in Scheme 3.

**Scheme 4:**
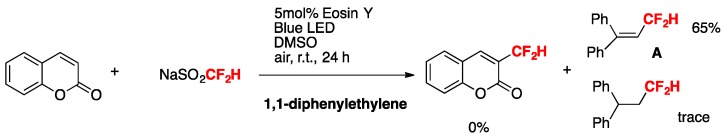
Radical trap experiments.

To gain some mechanistic insight into the reaction, the radical scavenger 2,2,6,6-tetramethyl-1-piperidinoxyl (TEMPO, 2 equiv) was added to the standard reaction mixture of coumarin and NaSO_2_CF_2_H. No desired product was formed. 1,1-diphenylethylene (2 equiv) was added to the standard reactions. Product A was encountered in a 65% yield. No coumarin-derived product was found (Scheme 4).

**Scheme 5:**
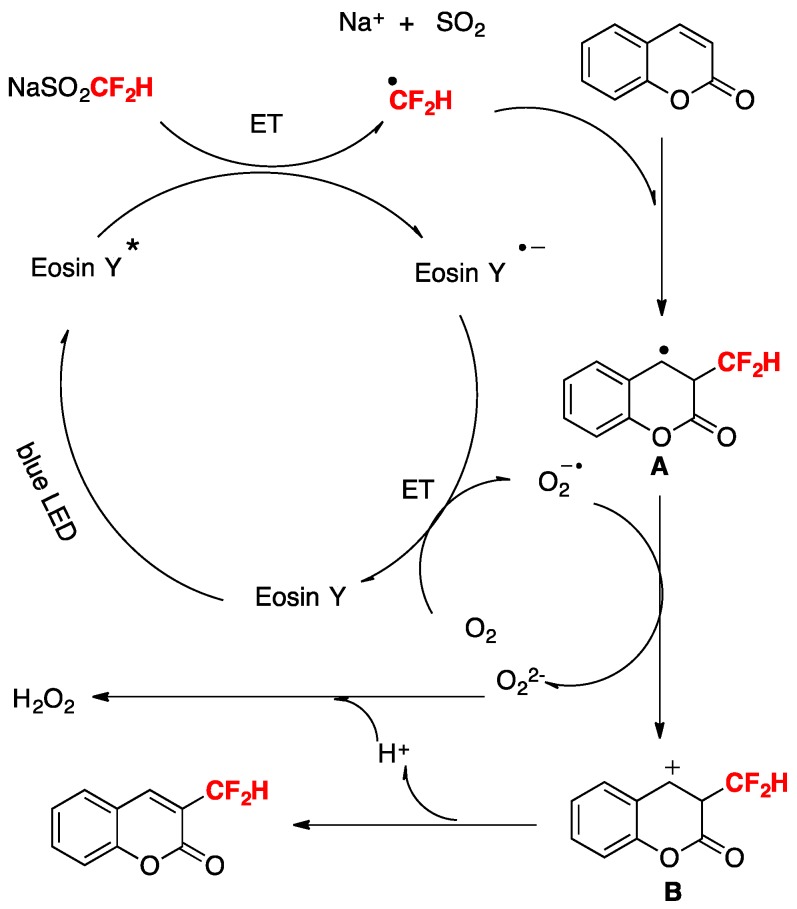
Proposed reaction mechanism.

Based on the previous control experiments, the authors in [36] proposed the reaction mechanism illustrated in Scheme 5. First, the excited Eosin Y* is formed under irradiation, and then a single electron is transferred from NaSO_2_CF_2_H to Eosin Y*, which generates EosinY^•−^ species and a highly reactive CF_2_H radical. Subsequently, radical intermediate A is generated. Then, one electron is transferred from Eosin Y^•−^ to O_2_ to give O_2_^•−^, regenerating the photocatalyst Eosin Y in its ground state. O_2_^•−^ is then further reacted with A to form O_2_^2−^ and intermediate B through an electron transfer sequence. Finally, deprotonation of this species furnishes the desired product (Scheme 5).

The regioselectivity of the difluoromethylation reaction of coumarin derivatives is driven by the stability of the benzyl radical obtained as intermediate.

Difluoromethylphosphonate BrCF_2_P(O)(OR)_2_ is an isostere-electronic phosphate mimic in vivo, and is a very important type of fluorinated reagent. The interest in the difluoromethylphosphonate reagent arises from the phosphonic acid functional groups in the CF_2_ motif, which mimics the tetrahedral transition state in peptide hydrolysis and enables these difluoromethylphosphonate-containing molecules to be used as phosphatase inhibitors.

**Scheme 6:**
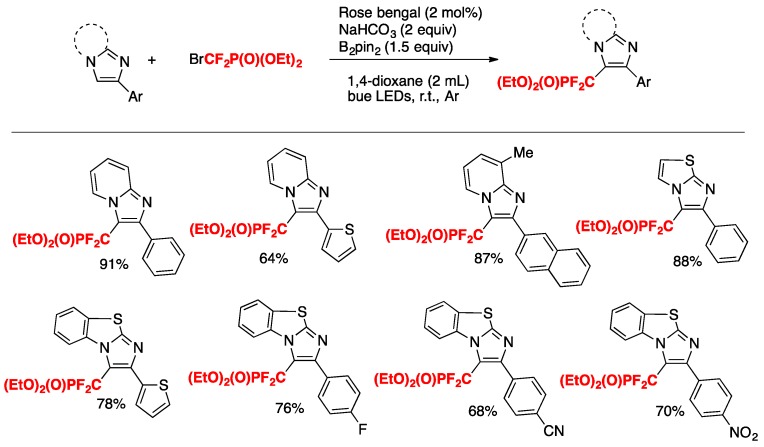
Selected examples for the difluoromethylenephosphonation of imodazoheterocycles.

Singsardar, Hajra, and collaborators [37] have recently accomplished the difluoromethylenephosphonation of imidazoheterocycles employing Rose Bengal (RB) as the photocatalyst, NaHCO_3_ as the base, B_2_pin_2_ as an additive in 1,4-dioxane as the solvent, and irradiated with blue LEDs, according to Scheme 6.

**Scheme 7:**
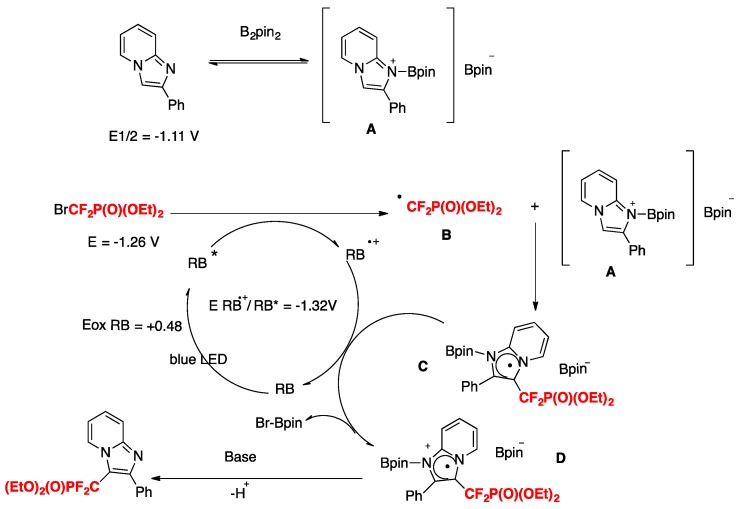
Proposed reaction mechanism.

In order to explore the mechanism, the authors in [37] carried out a series of control experiments. In the presence of TEMPO, 2,6-di*tert*-butyl-4-methyl-phenol, and *p*-benzoquinone, the reactions are completely suppressed. The addition of 1,1-diphenylethylene affords diethyl(1,1-difluoro-3,3-diphenylallyl)phosphonate. These observations point to a radical mechanism. The coupling product was not formed in the absence of B_2_pin_2_, suggesting that this additive stabilizes the imidazopyridine in the reaction medium. Based on these results, the authors in [37] proposed a reaction mechanism such as that shown in Scheme 7.

Initially, B_2_pin_2_ coordinates 2-phenylimidazo[1,2-a]pyridine by forming the reactive intermediate A. Under blue LED irradiation, RB is excited to its triplet state RB*. Following this, by ET from RB* to BrCF_2_PO(OEt)_2_, the CF_2_PO(OEt)_2_ radical species B is formed, along with the oxidized RB radical cation. Subsequently, the CF_2_PO(OEt)_2_ radical reacts with intermediate A to produce the radical intermediate C. Then, the radical intermediate C is further oxidized to intermediate D via regeneration of RB. Finally, abstraction of a proton by base from intermediate D affords the desired product. In the imidazo heterocycles, the more electron-rich position is the 3-position.

**Scheme 8:**
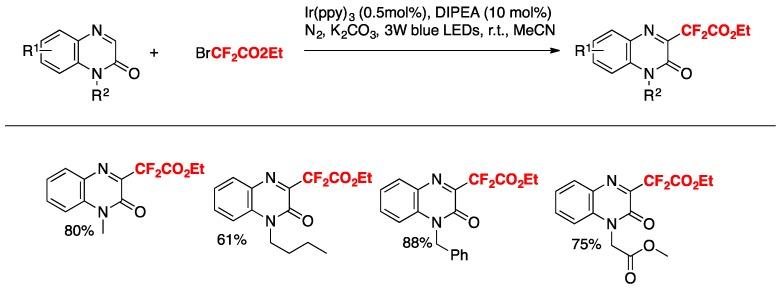
Selected examples for the difluoromethylation reaction of the quinoxalin-2(1*H*)-ones.

Jin, Sun, and colleagues [38] have recently accomplished the difluoromethylation of quinoxalin-2(1*H*)-ones employing *fac*-Ir(ppy)_3_ as the photocatalyst, BrCF_2_CO_2_R, as the difluoromethylating species in the presence of DIPEA and K_2_CO_3_ as bases in MeCN as the solvent, irradiated with blue LEDs. The scope of the reaction is illustrated in Scheme 8.

**Scheme 9:**
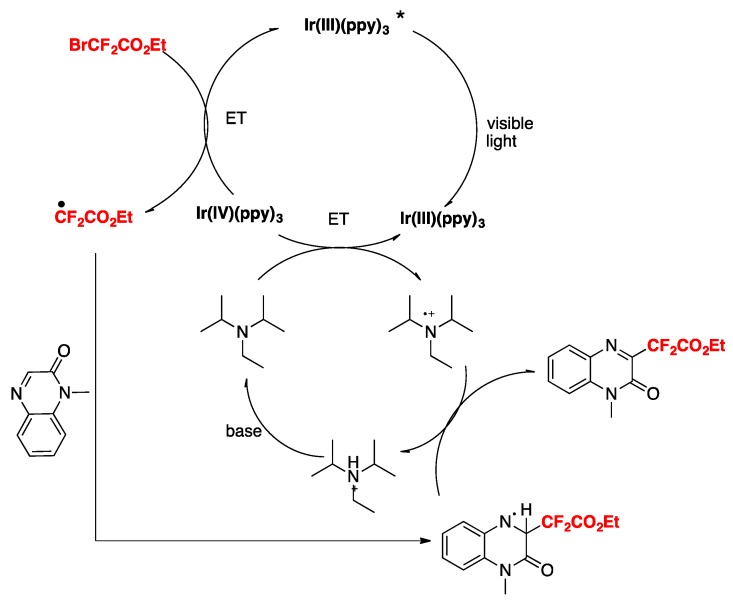
Proposed reaction mechanism.

The authors in [38] investigated the reaction mechanism employing radical probes such as BHT, 1,1-diphenylethylene, and TEMPO, not observing difluoromethylation of the quinoxalin-2(1*H*)-ones in all three cases. These sets of probe experiments seem to demonstrate the presence of radicals. The authors proposed an oxidative photocatalytic cycle of the Ir(ppy)_3_ photocatalyst, which from its excited state is able to reduce BrCF_2_CO_2_Et to CF_2_CO_2_Et radicals, rendering the photocatalyst into its oxidative Ir(IV) state. The Ir(III) photocatalyst is regenerated from the oxidized Ir(IV) by means of the DIPEA, which in turn is oxidized to its radical cation (DIPEA radical cation). The CF_2_CO_2_Et radicals in turn, add to the 3-position of the quinoxalin-2(1*H*)-ones, affording radical adducts of the CF_2_-substituted quinoxalin-2(1*H*)-ones, which are oxidized by the radical cation of DIPEA, closing the catalytic cycle (Scheme 9).

**Scheme 10:**
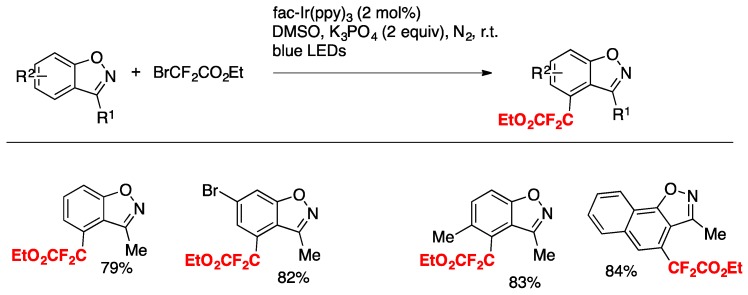
Selected examples for the difluoroalkylation of unactivated benzo[*d*]isoxazoles.

Hua, Xiong, Zhang, and colleagues [39] have accomplished the difluoroalkylation of inactivated benzo[*d*]isoxazoles with ethyl difluorobromoacetate by visible-light photoredox catalysis (*fac*-Ir(ppy)_3_) in the presence of K_3_PO_4_ as the base, in DMSO as the solvent, and irradiated with blue LEDs via direct and regioselective C–H functionalization (Scheme 10).

The mechanism proposed involves an oxidative quenching cycle of the photocatalyst, similar to other Ir(III) photocatalysis. The 4-position of the benzo[*d*]isoxazoles shown are those where substitution with the difluoroacetate radical takes place.

**Scheme 11:**
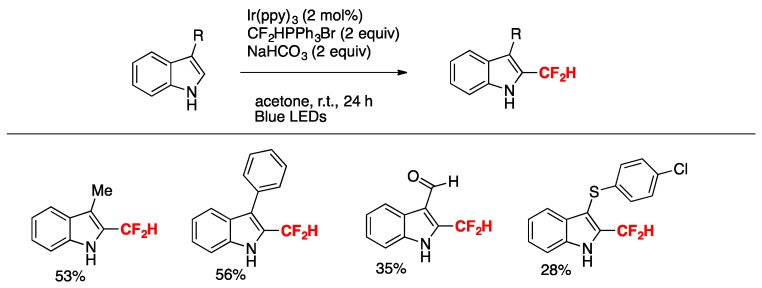
Selected examples for the 3-substituted indoles.

Rubisnki, Dolbier, and colleagues [40] attained the difluoromethylation of indoles under Ir(ppy)_3_ photocatalysis employing CF_2_HPPh_3_Br as a source of the difluoromethyl group in acetone as the solvent, and under blue LEDs irradiation in the presence of NaHCO_3_ as base. The scope of the reaction is succinctly summarized in Scheme 11.

### 2.2. CF_2_H Ipso Substitution of (Hetero)aromatic Compounds

In a seminal report, Bacauanu, MacMillan, and colleagues [41] applied the metallaphotoredox-catalyzed difluoromethylation of a wide array of aryl and heteroaryl halides employing commercially available CF_2_HBr as a source of CF_2_H radicals, a Ni catalyst, and a silane to act as a silyl-radical-mediated cross-electrophile coupling. The driving force is given by the low HF_2_C–Br-bond dissociation energy (BDE _HF2C-Br_ = 69 kcal mol^−1^) being far weaker than the Si–Br bond in a typical abstraction product (BDE _Me3Si-Br_ = 96 kcal mol^−1^) [42]. Moreover, given the electron-rich character of the silyl radical, the authors in [41] surmised that halogen abstraction from CF_2_HBr would be polarity-matched and hence kinetically faster than from previously utilized alkyl bromide substrates [43].

**Scheme 12:**
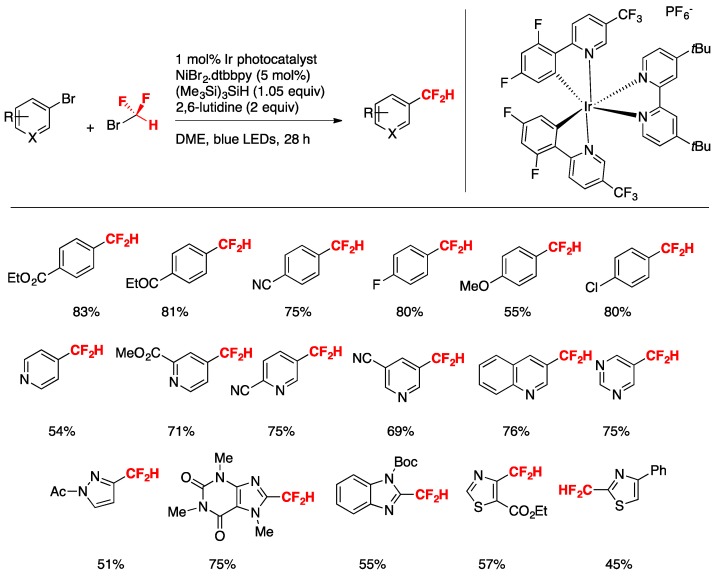
Scope of the silyl-radical-mediated difluoromethylation of aryl halide electrophiles using bromodifluoromethane as the CF_2_H source.

After carefully evaluating the conditions, the authors in [41] chose to conduct the reactions employing an aryl or heteroaryl substrate (0.5 mmol), Ir^III^ photocatalyst [Ir(dF(CF_3_)ppy)_2_(dtbbpy)]PF_6_ (1 mol%), NiBr_2_-dtbbpy (5 mol%), (Me_3_Si)_3_SiH (1.05 equiv), 2,6-lutidine as base (2 equiv), DME as the solvent, and irradiated with blue LEDs for 18 h. The scope of the transformation with respect to (hetero)arenes is illustrated in Scheme 12.

**Scheme 13:**
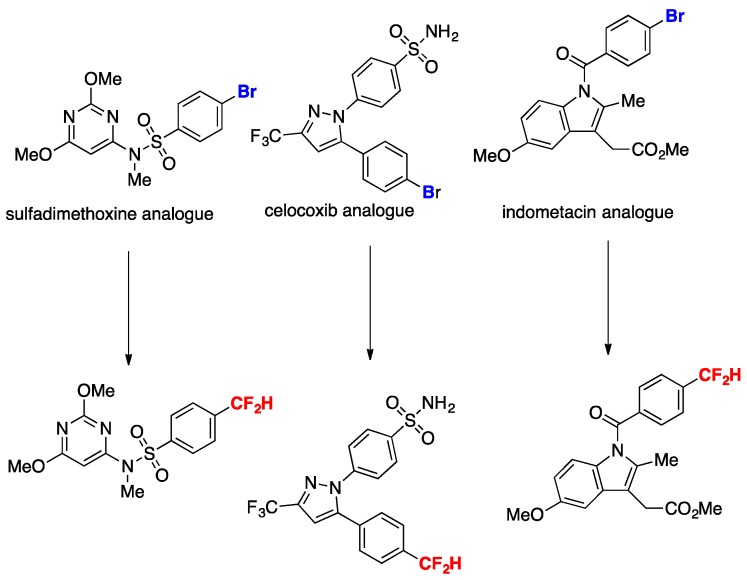
Late-stage functionalization for the expedient synthesis of difluoromethyl analogues of pharmaceutical agents.

Bromoarenes with electron-withdrawing groups afford good yields of *ipso* substitution products as well as bromoarenes with electron-donating groups. The authors in [41] applied the methodology to the late-stage functionalization of bioactive compounds such as those shown in Scheme 13, obtaining high yields of the respective difluoromethylated analogs, demonstrating the applicability of the transformation to substrates of biological relevance.

**Scheme 14:**
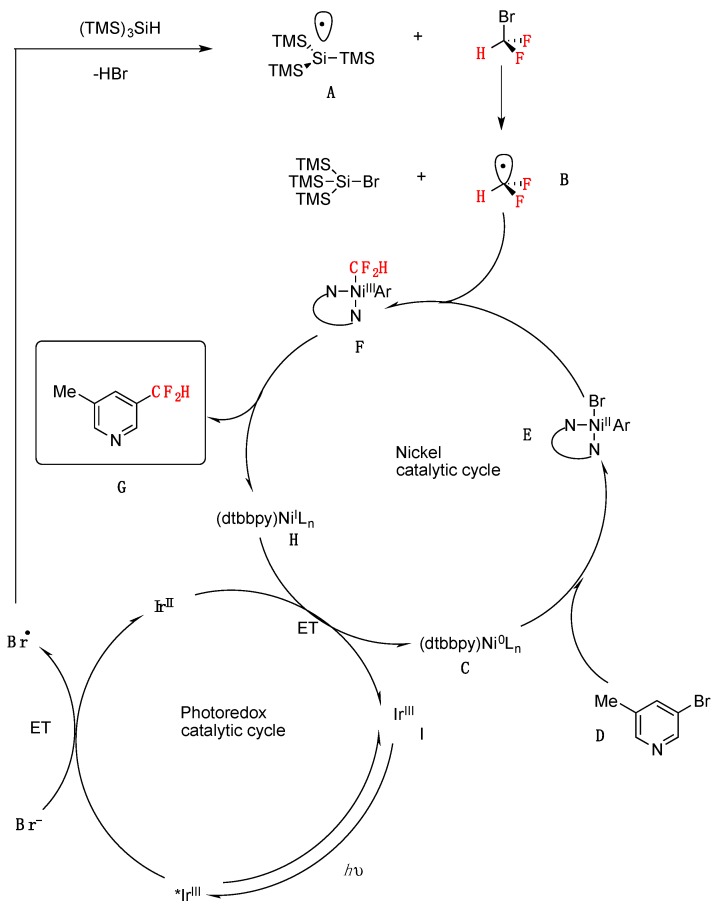
Proposed mechanism for metallaphotoredox-catalyzed difluoromethylation of aryl bromides.

The authors in [41] proposed a mechanism such as that illustrated in Scheme 14. Visible-light irradiation of Ir^III^ photocatalyst ([Ir(dF(CF_3_)ppy)_2_(dtbbpy)]PF_6_) generates the excited-state *Ir^III^ complex, which can readily oxidize bromide anion (E_1/2 red_[*Ir^III^/Ir^II^] = + 1.21 V vs. SCE in MeCN; E_1/2 red_[Br^.^/Br^−^] = + 0.80 V vs. SCE in DME) [44]. The resulting bromine radical can participate in hydrogen atom transfer with (Me_3_Si)_3_SiH to yield the nucleophilic silyl radical A [45]. Bromine atom abstraction from BrCF_2_H (162) by open-shell silyl species A would then afford the key difluoromethyl radical intermediate (B). Along with the photoredox catalytic cycle, Ni^0^ catalyst C [46] is expected to suffer facile oxidative addition into aryl bromide D to generate Ni^II^–aryl intermediate E. Trapping of CF_2_H radical (B) would then generate the corresponding aryl–Ni^III^–CF_2_H complex F, which upon reductive elimination should afford the desired difluoromethylarene product G and Ni^I^ species H. Finally, an electron transfer between H and reduced photocatalyst would simultaneously regenerate low-valent nickel catalyst C and ground-state photocatalyst I (Scheme 14).

## 3. Photocatalytic Difluoromethylation of Multiple Bonds

### 3.1. Difluoromethylation of Aliphatic Carbon–Carbon Multiple Bonds

**Scheme 15:**
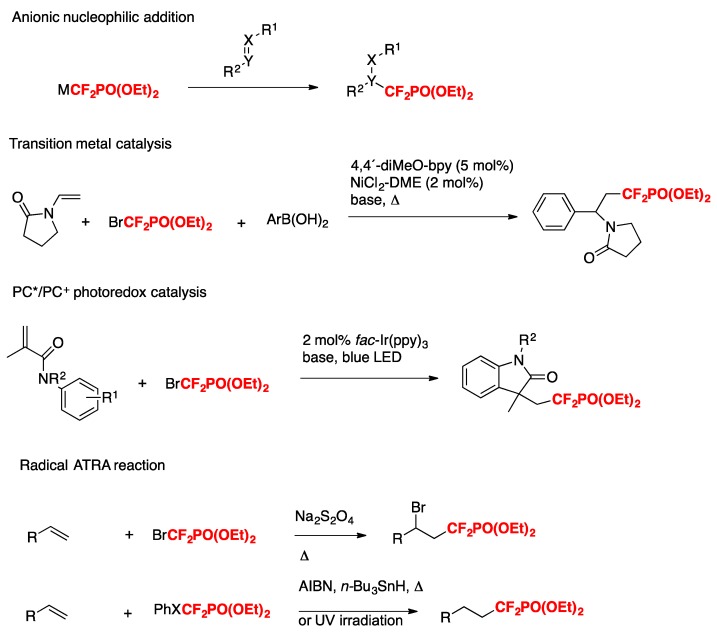
Strategies for constructing sp^3^ C−CF_2_PO(OEt)_2_ bonds.

The phosphonodifluoromethylation of alkenes has been documented to proceed through diverse methods such as nucleophilic addition [47,48,49,50,51,52,53,54], transition metal catalysis [55,56,57,58], photoredox catalysis (by oxidative quenching cycles) [59,60,61,62,63], or radical ATRA reactions [64,65,66,67,68,69], as depicted in Scheme 15.

In general, terminal olefinic carbons are the reactive positions of difluoromethyl radicals and many more odd-electron reactive intermediates.

**Scheme 16:**
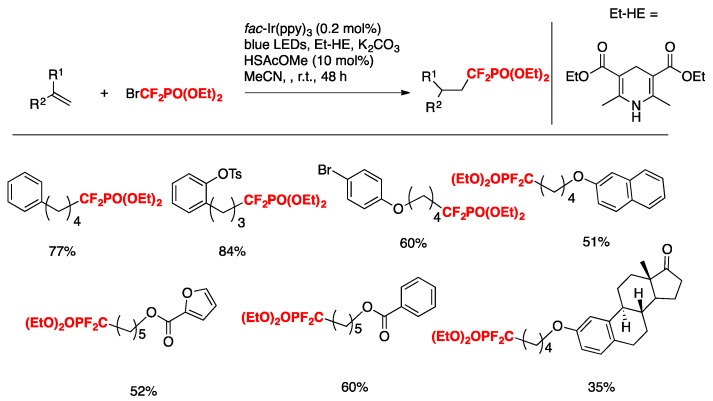
Selected examples for the hydrophosphonodifluoromethylation of mono- and disubstituted alkenes.

Huang, Chen, Tiang, and colleagues [70] have recently accomplished the visible-light-induced catalytic hydrophosphonodifluoromethylation of mono- and disubstituted alkenes using BrCF_2_PO(OEt)_2_ with a Hantzsch ester as the terminal reductant. The combination of thiyl-radical catalysis with photoredox catalysis is important for achieving good chemoselectivity and high yields. The optimized conditions involve the alkene substrate (0.1 mmol), BrCF_2_PO(OEt)_2_ (2 equiv), *fac-*Ir(ppy)_3_ (0.2 mol %), HSAcOMe (10 mol %), Hantzsch ester (EtHE, 1.5 equiv), blue LEDs, in MeCN as the solvent (1 mL) at room temperature for 48 h. The scope of the transformation is illustrated in Scheme 16.

**Scheme 17:**
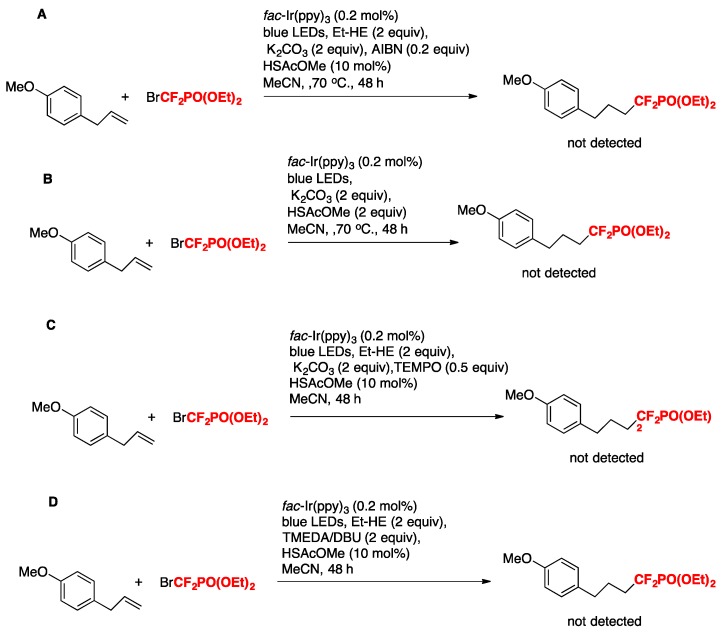
Control experiments. **A** reaction in the presence of AIBN. **B** reaction in the presence of HSAcOMe. **C** reaction in the presence of TEMPO. **D** reaction in the presence of TMEDA/DBU.

The authors in [70] carried out control experiments in order to elucidate the reaction mechanism. A reaction between alkene and BrCF_2_PO(OEt)_2_ in the presence of 2,2′-azobis(2-methylpropionitrile) (AIBN) as a radical initiator showed no conversion of alkene, implying that an electron transfer process is essential for the transformation (Scheme 17A). When HSAcOMe (2 equiv) was used as the sole hydrogen source (Et–HE was omitted), no product was detected, implying that this thiol and related species were not reductive enough to drive this transformation (Scheme 17B). The authors in [70] found that 0.5 equiv of TEMPO completely quenched the reaction, suggesting that a radical reaction pathway was involved (Scheme 17C). In addition, when an even more reductive tertiary amine such as TMEDA (E_1/2_ red = + 0.72 vs. SCE)/DBU) was used instead of Et–HE (E_1/2_ red = + 0.92 V vs. SCE), the reaction provided none of the expected product (Scheme 17D). In addition, the sluggishness of the reaction in the absence of HSAcOMe suggests the importance of the thiol for the generation of the Ir^II^ species.

**Scheme 18:**
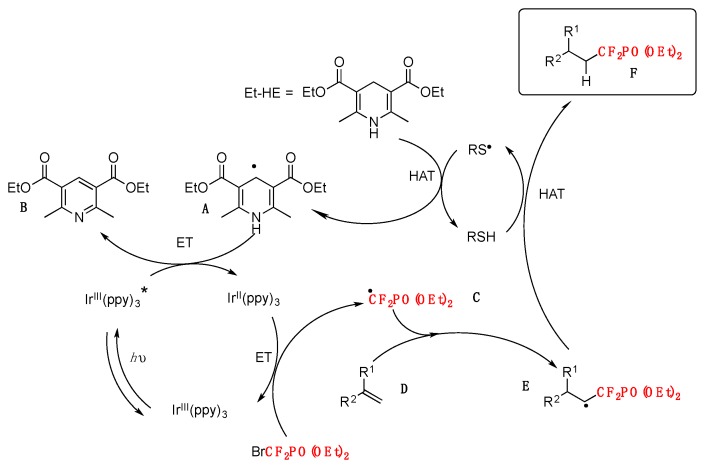
Plausible reaction pathway.

Based on the above and other experiments, the authors in [70] postulate a mechanism such as that shown in Scheme 18.

First, a thiyl radical RS^•^ generated from methyl 2-mercaptoacetate HSAcOMe abstracts a hydrogen atom from the Hantszch ester Et–HE, giving rise to a thiol and Et–HE radical A. Radical A is sufficiently reductive (E_1/2_ red < −1.15 V vs. SCE) to transfer a single electron to the excited-state of Ir* (E_1/2_ Ir^III^*^/II^ = 0.31 V vs. SCE), which results in the formation of a pyridine compound B and *fac*-Ir ^II^(ppy)_3_. Subsequently, radical C, which is formed from BrCF_2_PO(OEt)_2_ (E_1/2_ red = −1.74 V vs. SCE) and Ir^II^ (E_1/2_ Ir^III/II^ = −2.19 V vs. SCE) via another single-electron transfer, adds to the alkene D to produce radical intermediate E. As no Ir^IV^ species is present at this stage, the radical does not undergo oxidation. Finally, hydrogen atom transfer between E and the thiol RSH furnishes product **F**. 

**Scheme 19:**
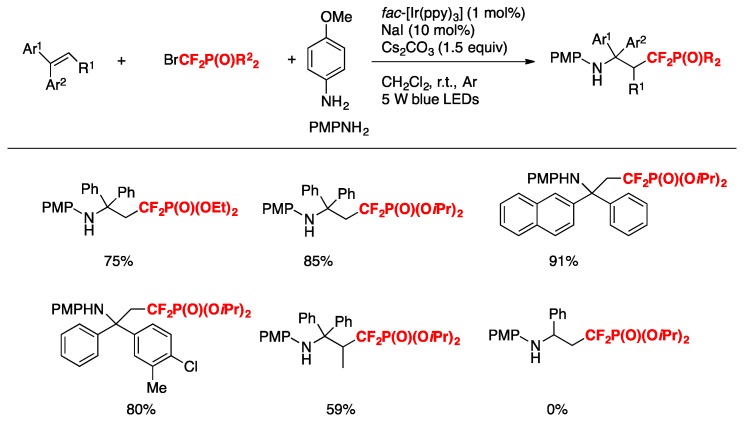
Selected examples for the synthesis of bromodifluoromethylphosphonates from alkenes.

Q. Yang, S.-D. Yang, and collaborators [71] have disclosed an efficient and practical method for the synthesis of α,α-difluoro-γ-aminophosphonates through photocatalyzed intermolecular aminodifluoromethylphosphonation of alkenes utilizing BrCF_2_P(O)(OR)_2_ as the difluoromethylating reagent. The authors optimized the reaction conditions employing 1,1-diphenylethene (0.2 mmol), BrCF_2_P(O)(OEt)_2_ (0.4 mmol), *p*-amino-anisol (0.28 mmol), *fac*-[Ir(ppy)_3_] (1 mol%), NaI (10 mol %), Cs_2_CO_3_ (0.3 mmol) in CH_2_Cl_2_ as the solvent, for 24 h under blue LED irradiation. The substrate scope of alkenes and bromodifluoromethylphosphonates is illustrated in Scheme 19.

The authors in [71] also investigated the substrate scope with regard to the amine, employing different nitrogen bases, affording good results of the aminodifluoromethylphosphonation products derived from 1,1-diphenylethene.

**Scheme 20:**
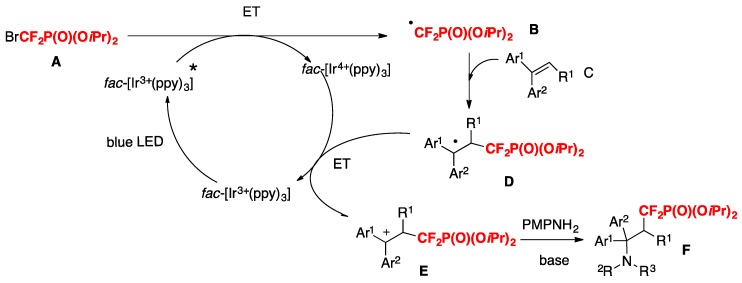
Plausible mechanism.

The authors in [71] proposed a reaction mechanism such as that shown in Scheme 20. First, the photocatalyst *fac-*[Ir(ppy)_3_] is excited by visible light irradiation (blue LEDs) to generate the excited Ir ^3+*^, which could undergo an ET process with the difluorine reagent BrCF_2_P(O)(O*i*Pr)_2_ **A** to form the difluoromethylphosphonation radical **B** and Ir^4+^. The electrophilic radical **B** adds to alkenes **C** to form intermediate **D**, which was subsequently oxidized to the intermediate **E** by Ir^4+^. Finally, the intermediate **E** is attacked by nucleophilic arylamines (PMPNH_2_) to afford the cation intermediate, followed by base-mediated deprotonation to give the final product **F.**

**Figure 3:**
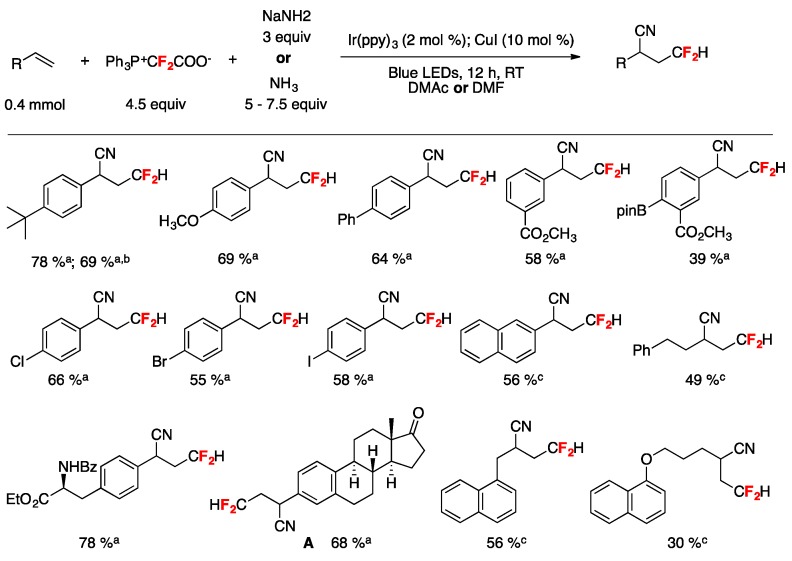
Substrate scope examples for the photocatalyzed cyanodifluoromethylation of alkenes [72]. [a] Using NaNH2 and DMAc (*N,N*-dimethyl acetamide) as the solvent; [b] gram scale reaction; [c] using NH3 and DMF (*N,N*-dimethylformamide) as the solvent.

**Scheme 21:**
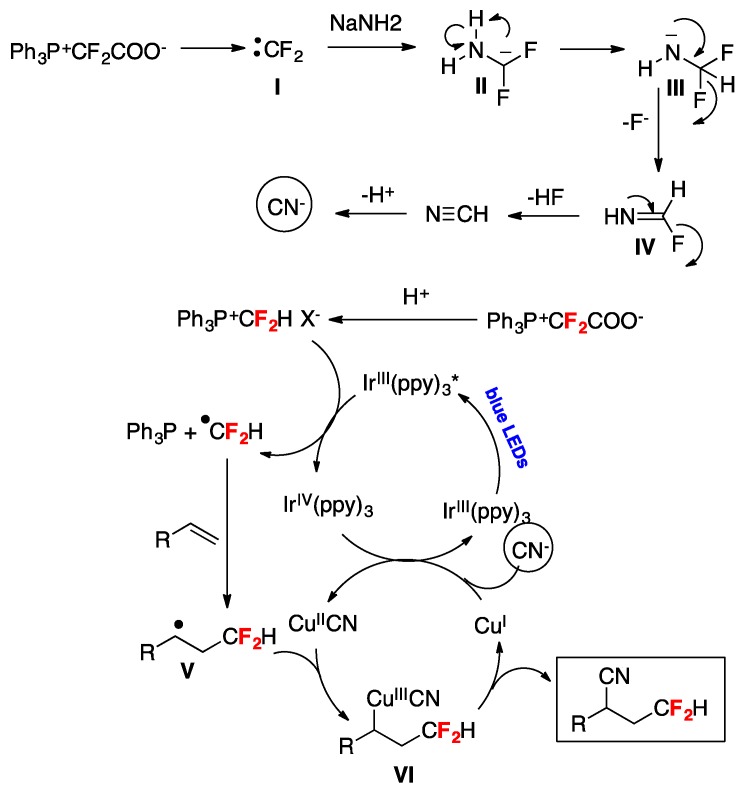
Proposed reaction mechanism for the photocatalyzed cyanodifluoromethylation of alkenes [72].

A synthetic photocatalyzed methodology for the cyanodifluoromethylation of alkenes has been developed by Xiao and collaborators [72]. The reactions employ a Ph_3_P^+^CF_2_CO_2_^−^/NaNH_2_ (or NH_3_) reagent system where Ph_3_P^+^CF_2_CO_2_^−^ functions as the CN and the HCF_2_ source. Optimum conditions were reached employing 4-*tert*-butyl styrene as the substrate by means of *tris*-(2-phenylpyridine)-iridium (Ir(ppy)_3_) photocatalysis under blue LEDs irradiation in the presence of Ph_3_P^+^CF_2_CO_2_^−^/NaNH_2_ (or NH_3_) and CuI using dimethylacetamide (DMA) or dimethylformamide (DMF) as the solvent under an Ar atmosphere. With the optimized reaction conditions in hand, the authors in [72] applied the methodology to various aryl alkenes, proving that neither electron-withdrawing nor -donating groups have a significant impact on the reaction yield; a variety of functional groups including ketone, esters, iodide, bromine, chloride, boronic acid ester, amide, and ether were well tolerated (Figure 3). The synthetic utility of this method was further demonstrated by the synthesis of a cyanodifluoromethylated estrone derivative A (Figure 3) [72]. It should be noted that in addition to aryl alkenes, alkyl-substituted alkenes also afforded the expected reaction products (Figure 3) [72]. Scheme 21 shows, through the proposed reaction mechanism, how Ph_3_P^+^CF_2_CO_2_^−^ serves as both the CN and the HCF_2_ source; initially a difluorocarbene **I**, generated by decomposition of Ph_3_P^+^CF_2_CO_2_^−^, is trapped by NaNH_2_ affording carbanion **II** that through hydrogen migration from the N–H moiety to the carbon anion yields intermediate **III**, which by fluorine elimination gives formidoyl fluoride **IV** (Scheme 21). Upon formidoyl fluoride further F-elimination and deprotonation, HCN is produced and deprotonation ensues the cyanide anion (Scheme 21). The authors proposed that the phosphonium salt Ph_3_P^+^CF_2_HX^−^ generated by protonation of Ph_3_P^+^CF_2_CO_2_^−^ is reduced by the photoexcited photocatalyst generating an Ir^IV^ complex and HCF_2_^•^ radicals, which are captured by the alkene substrate affording the carbon centered radical adduct **V** (Scheme 21). The Ir^IV^ complex is an oxidant species and oxidizes Cu^I^ to Cu^II^ (regenerating the Ir(ppy)_3_ photocatalyst) and through a rapid ligand exchange affords the Cu^II^CN complex, which by reaction with radical adduct **V** produces Cu^III^ complex **VI**. Reductive elimination of Cu^III^ complex **VI** affords the final product regenerating the Cu^I^ catalyst (Scheme 21) [72].

**Figure 4:**
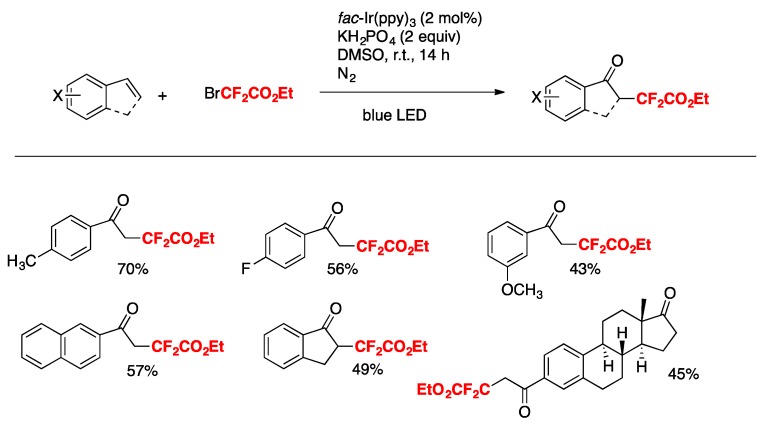
Selected examples for the visible-light promoted oxo-difluoroalkylation of alkenes with dimethyl sulfoxide.

More recently, Xia, Ye, and colleagues [73] have accomplished the visible-light promoted oxo-difluoroalkylation of alkenes with dimethyl sulfoxide as the solvent and oxidant. This procedure affords the corresponding α,α-difluoro-γ-ketoacetates. The scope of the transformation is illustrated in Figure 4.

**Scheme 22:**
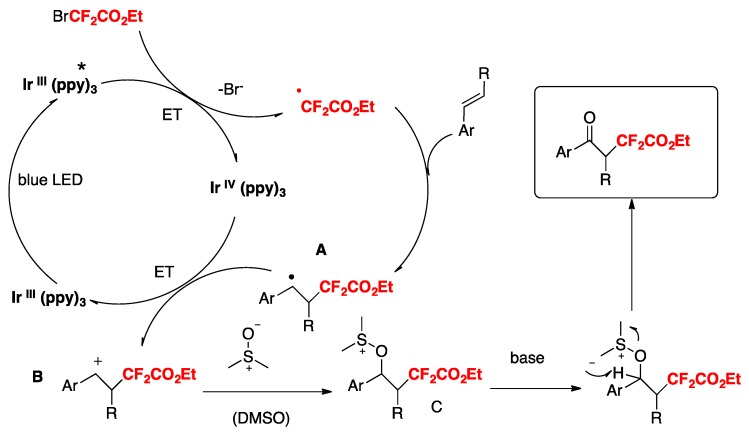
Proposed reaction mechanism.

Control experiments reveal that the reaction is radical in nature (absence of product in the presence of TEMPO). When the reaction is carried out in the presence of tetrahydrothiophene-1-oxide instead of DMSO, the corresponding α,α-difluoro-γ-ketoacetates are also obtained. A likely mechanism is illustrated in Scheme 22. Under visible light irradiation, excited ***fac-*Ir(ppy)*_3_*** reduces BrCF**_2_**CO**_2_**Et to produce CF**_2_**CO**_2_**Et radicals (and Ir(IV)). Addition of CF_2_CO_2_Et radicals to terminal alkene carbon affords intermediate A, which is oxidized by Ir(IV) to intermediate B (regenerating Ir(III). Intermediate B undergoes a Korblum oxidation with DMSO to yield intermediate C, which by base-promoted elimination gives the product.

Continuous-flow chemistry has recently attracted significant interest from chemists in both academia and industry working in different disciplines and from different backgrounds. Flow methods are now being used in reaction discovery/methodology, in scale-up and production, and for rapid screening and optimization. Photocatalyzed processes carried out in flow systems are currently an important research field in the scientific community and the recent exploitation of flow methods for these methodologies has made clear the advantages of flow chemistry and its importance in modern chemistry and technology worldwide [74].

More recently, Nakayama, Koike, Akita, and colleagues [75] have accomplished a step-economical method for the synthesis of α- CF_2_H-substituted ketones from readily available alkenes. Electrochemical analysis, laser flash photolysis (LFP), and density functional theory (DFT) calculations reveal that *N-*tosyl-*S*-difluoromethyl-*S*-phenylsulfoximine serves as the best CF_2_H radical source among analogous sulfone-based CF_2_H reagents.

**Scheme 23:**
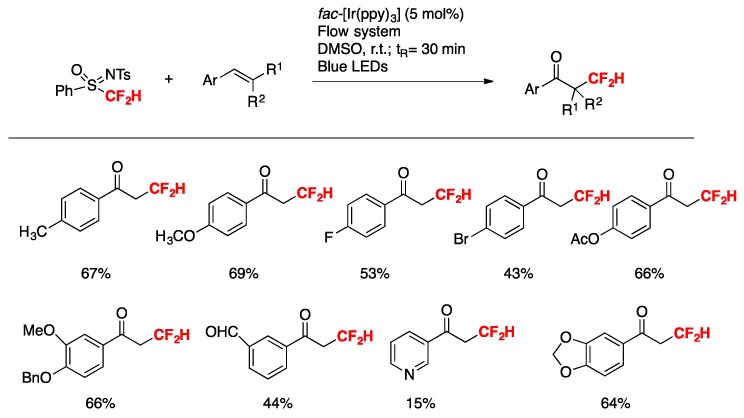
Selected examples for the synthesis of the keto-difluoromethylated styrene derivatives in flow.

The scope of the transformation is illustrated in Scheme 23.

**Scheme 24:**
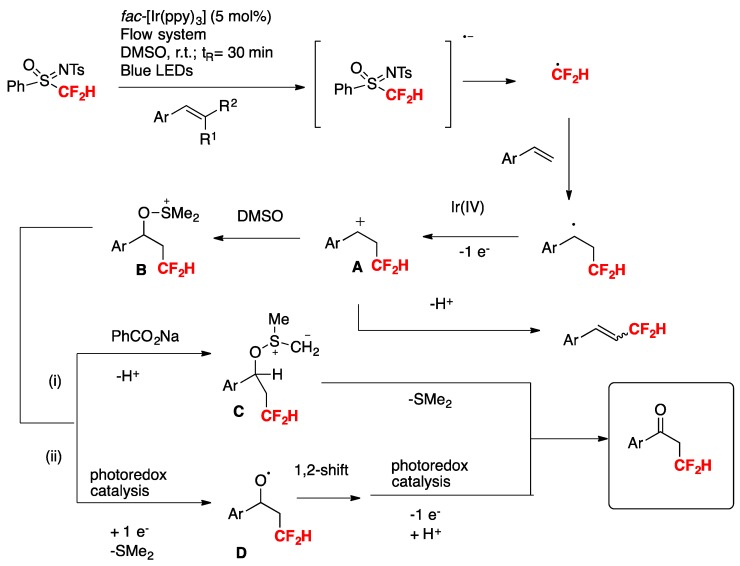
Proposed reaction mechanism for the synthesis of α-CF_2_H-substituted ketones.

Quantum yield determinations reveal that the photocatalytic reaction of 4-methylstyrene with *N*-tosyl-*S*-difluoromethyl-*S*-phenylsulfoximine (PhS(O)NTsCF_2_H) reagent does not show a chain propagation event, as the quantum yield value determined for the photocatalytic reaction was 0.28. The authors in [75,76,77] proposed a reaction mechanism as depicted in Scheme 24.

To commence with, photoexcitation of the photocatalyst *fac-*[Ir(ppy)_3_] renders the excited triplet state of Ir(III)*, which reduces the *N*-tosyl-S-difluoromethyl-S-phenylsulfoximine to its radical anion that readily decomposes to CF_2_H radicals. CF_2_H radicals add to the terminal position of the olefin to give a radical adduct, who in turn is oxidized to benzyl cation **A** by the oxidized form of the photocatalyst (Ir(IV)). Cation A reacts with DMSO to render intermediate **B**. This intermediate **B** can in turn follow two reaction pathways: (i) and (ii). In pathway (i), deprotonation by the base affords intermediate **C,** which by ulterior loss of dimethylsulfide gives the final ketone product, or else, through step (ii), intermediate **B** can suffer photocatalyzed-induced reduction with concomitant loss of dimethylsulfide to render intermediate **D**. Upon 1,2-shift and reduction followed by deprotonation, intermediate **D** affords the final ketone product.

**Scheme 25:**
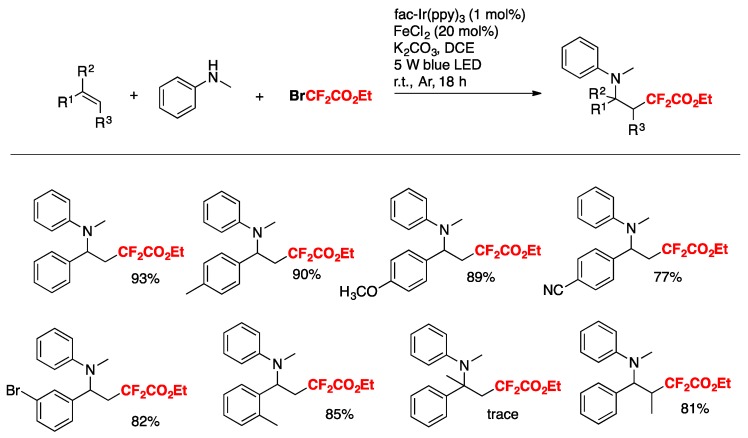
Selected examples of the visible-light photoredox cooperative catalyzed three-component 1,2-difluoroalkylamination of alkenes with BrCF**_2_**CO**_2_**Et and amines.

Xu and Cai [76] have recently accomplished the iron and visible-light photoredox cooperative catalyzed three-component 1,2-difluoroalkylamination of alkenes with BrCF**_2_**CO**_2_**Et and amines. The scope of the transformation is described in Scheme 25.

**Scheme 26:**
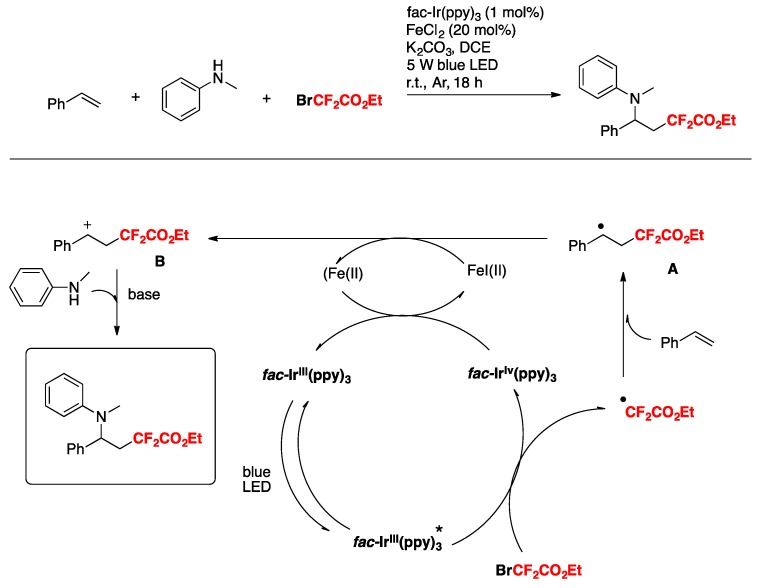
Proposed reaction mechanism for the cooperative catalyzed three-component 1,2-difluoroalkylamination of alkenes with BrCF_2_CO_2_Et and amines.

The authors also employed other amines such as 4-methyl-N-methylaniline, 4-methoxy-N-methylaniline, etc., obtaining good yields of the three-component products. The reaction in the presence of TEMPO, or hydroquinone, does not afford a product, purporting to the presence of radicals. On the basis of these results and literature precedents, the authors in [76] proposed a reaction mechanism such as that depicted in Scheme 26. First, irradiation of the catalyst *fac-*Ir(III) (ppy)_3_ affords an excited state species, which is a strong reductant of BrCF_2_CO_2_Et, giving CF_2_CO_2_Et radicals, which in turn add to the terminal olefinic position to give radical adduct A. Radical adduct A is oxidized by Fe(III), which in turn is produced by the Ir(IV)-mediated oxidation of Fe(II). Oxidation of radical adduct A ensues to carbocation B, which is attacked nucleophilically by the N-methylaniline. Upon base-deprotonation, the final three-component product is obtained.

**Scheme 27:**
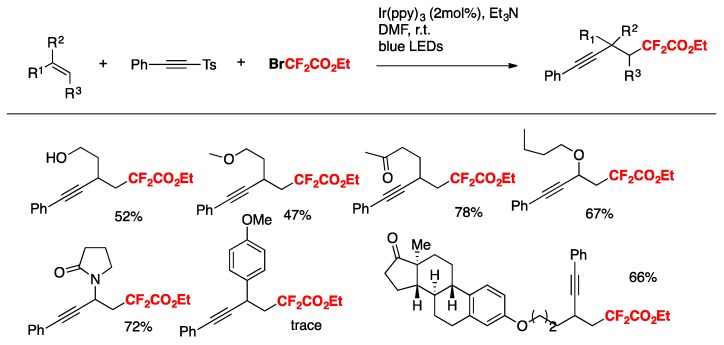
Selected examples for the visible-light induced three component reaction consisting of difluoroalkyl halides, unactivated alkenes, and alkynyl sulfones.

Jin, G. Zhu, and colleagues [77] informed a visible-light induced three component reaction consisting of difluoroalkyl halides, unactivated alkenes, and alkynyl sulfones. A scope of the transformation is summarized in Scheme 27.

**Scheme 28:**
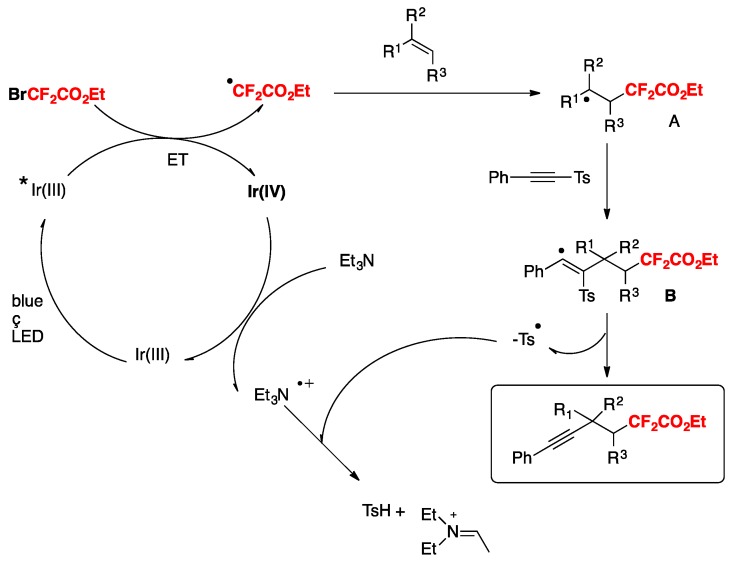
Proposed reaction mechanism for the visible-light induced three component reaction consisting of difluoroalkyl halides, unactivated alkenes, and alkynyl sulfones.

The authors in [77] also investigated the reaction with respect to the alkynyl sulfones, observing good scope with different sulfones. Inhibition of the reaction is accomplished by the use of TEMPO, indicating the presence of radicals. A plausible mechanism is proposed according to the latter and other mechanistic probe experiments, as illustrated in Scheme 28.

Radicals CF**_2_**CO**_2_**Et are generated from the ET-reduction from excited Ir(III). The CF_2_CO_2_Et radical adds to the less-impeded olefinic carbon to render intermediate A. Intermediate A adds to the sp carbon atom to give intermediate B, which by subsequent tosyl radical extrusion yields the final product.

### 3.2. Difluoromethylation of Carbon–Carbon Multiple Bonds and Ulterior Cyclization

**Scheme 29:**
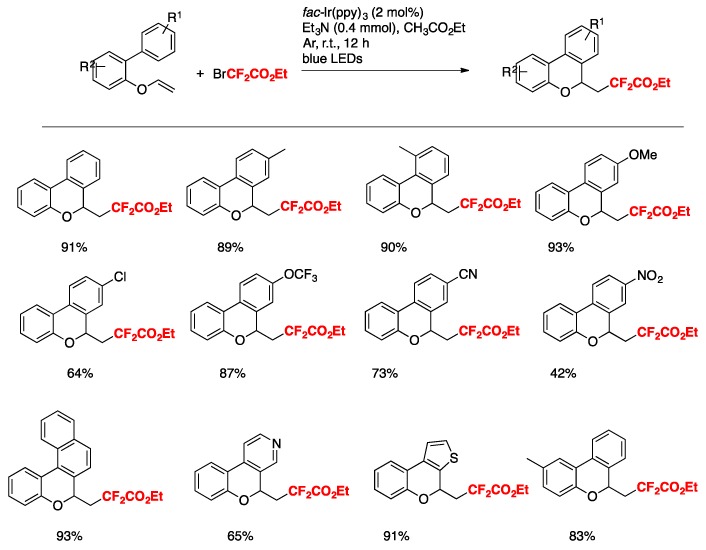
Selected examples for the addition/cyclization of biaryl vinyl ethers with ethyl 2-bromo-2,2-difluoroacetate.

Deng, Sun, and colleagues [78] have reported the visible-light-promoted cascade cyclization reaction for the synthesis of 6-fluoroalkyl 6*H*-benzo[*c*]-chromenes, which was initiated by intermolecular radical addition to biaryl vinyl ethers using easily available fluoroalkylated reagents such as BrCF_2_CO_2_Et or 2-bromo-2,2- difluoroamides as the sources of fluorinated radicals, followed by the cyclization onto an aromatic ring. The optimized reaction conditions employ biaryl vinyl ethers (0.2 mmol), BrCF_2_CO_2_Et or 2-bromo-2,2-difluoroamides (0.4 mmol), Et_3_N (0.4 mmol), and *fac*-Ir(ppy)_3_ (2 mol %) in CH_3_COOEt as the solvent (2 mL) under an Ar atmosphere upon irradiation of 5 W blue LEDs at room temperature for 12 h. The scope of the transformation using BrCF_2_CO_2_Et is illustrated in Scheme 29.

**Scheme 30:**
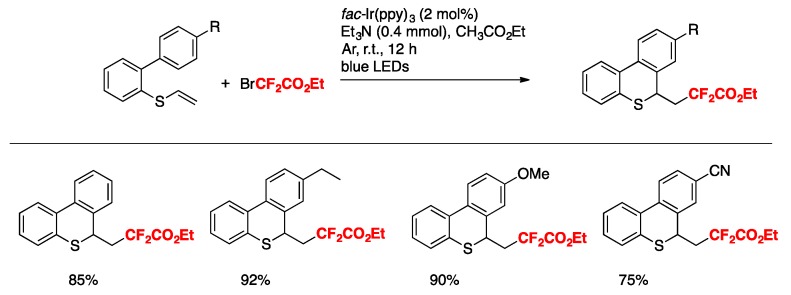
Addition/cyclization of biaryl vinyl thioethers with ethyl 2-bromo-2,2-difluoroacetate.

The authors in [78] also attempted the homologous vinyl thioethers as substrates, and the corresponding difluoroalkylated benzothiochromenes could also be obtained under the same reaction conditions. Some examples are depicted in Scheme 30.

**Scheme 31:**
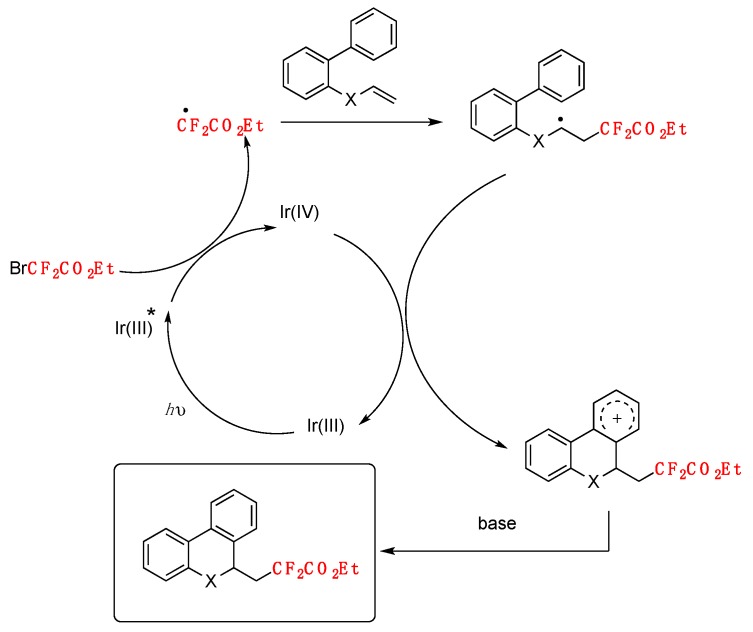
Proposed reaction mechanism.

To elucidate the mechanism of the reaction, several control experiments have been designed and carried out. When radical scavenger 2,2,6,6-tetramethylpiperidineoxy (TEMPO) was added to the reaction mixture, no product was formed. A radical coupling product was encountered in the presence of 1,1-diphenylethene. These results indicated that a radical pathway might be involved in the mechanism, and a radical addition to the carbon−carbon double bond might be prior to the cyclization process. In the absence of a photocatalyst, the reaction could not take place. Based on the above experiments, a mechanism was proposed by the authors in [78], according to Scheme 31.

**Scheme 32:**
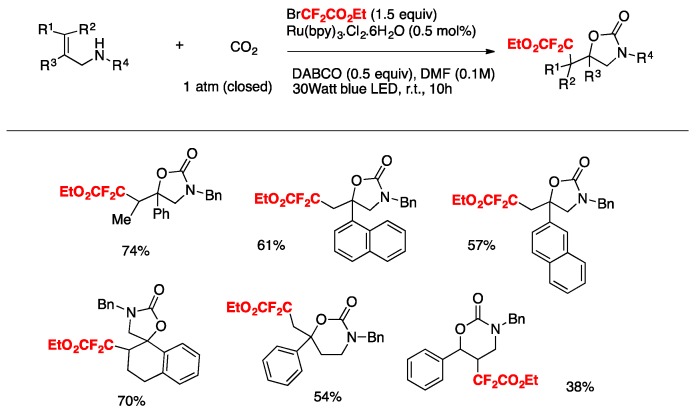
Selected examples for the oxy-difluoroalkylation of allylamines with CO_2_ via visible-light photoredox catalysis to generate a series of important 2-oxazolidinones with functionalized difluoroalkyl groups.

Yin, Yu, and colleagues [79] achieved the oxy-difluoroalkylation of allylamines with CO_2_ via visible-light photoredox catalysis to generate a series of relevant 2-oxazolidinones with functionalized difluoroalkyl groups employing BrCF_2_CO_2_Et as the source of radicals, Ru(bpy)_3_.Cl_2_.6H_2_O as the photocatalyst, DABCO as the base, DMF as the solvent, and irradiated with a blue LED. The scope employing allylamines is illustrated in Scheme 32.

**Scheme 33:**
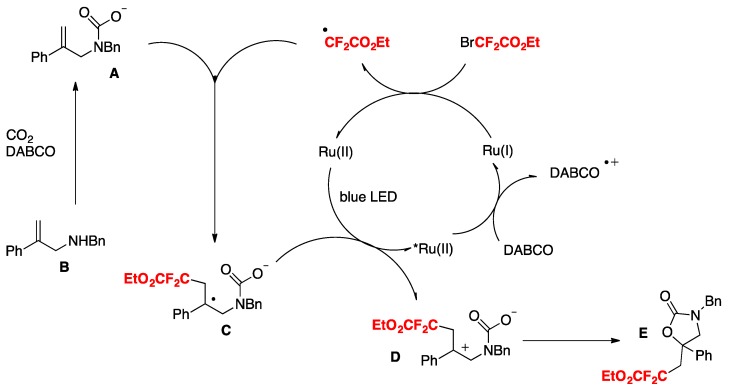
Proposed reaction mechanism for the oxy-difluoroalkylation of allylamines with CO_2_ via visible-light photoredox catalysis.

The authors in [79] advanced a proposal for the realization of the mechanism, as depicted in Scheme 33. Based on mechanistic probe studies, a proposed reductive quenching of excited photocatalyst by DABCO (Ep ox = + 0.69 V vs. SCE, E_[Ru(II)*/Ru(I)]_ = + 0.77 V) provides Ru(I), which reduces BrCF_2_COOEt to generate the CF_2_COOEt radical. Then, the addition of the CF_2_COOEt radical to carbamate **A** (Scheme 33), which is generated from **B** and CO_2_ in situ, gives benzylic radical **C**. Subsequent oxidation of **C** by excited Ru(II) species and intramolecular cyclization of **D** affords product **E**. Other pathways cannot be ruled out at this time.

**Figure 5:**
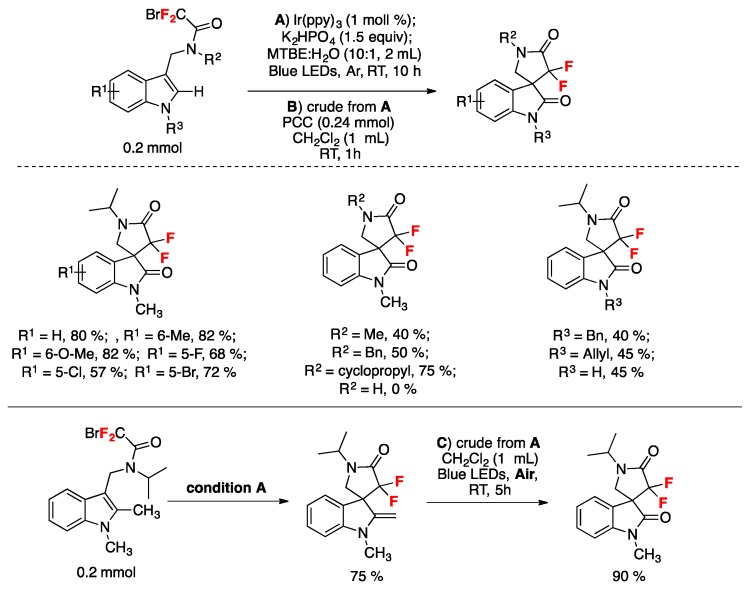
Substrate scope examples for the synthesis of *gem*-difluorinated spiro-γ-lactam oxindoles mediated by a sequential process involving visible light-induced consecutive difluoromethylative dearomatization, hydroxylation, and oxidation [80].

**Scheme 34:**
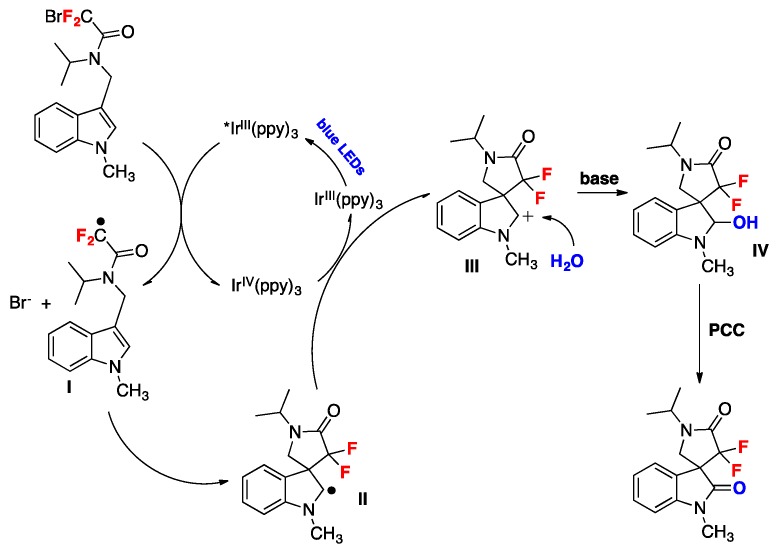
Proposed reaction mechanism for the synthesis of *gem*-difluorinated spiro-γ-lactam oxindoles mediated by a sequential process involving visible light-induced consecutive difluoromethylative dearomatization, hydroxylation, and oxidation.

Wang et al. described a protocol for the synthesis of *gem*-difluorinated *spiro*-γ-lactam oxindoles mediated by a sequential process involving visible light-induced consecutive difluoromethylative dearomatization, hydroxylation, and ulterior oxidation [80]. Optimized reaction conditions were established in a two-step one-pot reaction employing indole substrates substituted at C-3 with a bromodifluoroacetamide moiety. The first step involves a photocatalytic process with Ir(ppy)_3_, K_2_HPO_4_ as the base, in a mixture of methyl *tert*-butyl ether and water as the solvent using blue LED irradiation at room temperature under an Ar atmosphere (conditions A, Figure 5); this is followed by a second step that requires the addition of pyridinium chlorochromate (PCC) as an oxidant in CH_2_Cl_2_ as the solvent (conditions B, Figure 5). For C-2-substituted indole substrates, reaction with PCC was not required and, in these cases, the crude reaction mixture containing the methylene products were exposed to air and irradiated with blue LEDs in CH_2_Cl_2_ as the solvent (conditions C, Figure 5). The reaction proved to be useful, affording moderate to very good yields of products with a variety of substrates bearing different substituents. Compounds with electron-donating or electron-withdrawing substituents on the indole ring (R^1^, Figure 5) afforded the corresponding products. Studies on the amide moiety evidenced that the reaction was sensitive to the substituent on the amide nitrogen (R^2^, Figure 5); the desired product was not afforded with R^2^ = H; this is probably explained by the easy oxidation of this compound. Then, the authors in [80] focused their attention on the nitrogen of the indole moiety (R^3^, Figure 5), and obtained the desired reaction products when R^3^ was a benzyl or allyl substituent, or even a H atom. C-2-substituted indoles also reacted under conditions A (Figure 5), even though the reaction yielded a methylene product, presumably via a s β-H elimination involving the C-2 cation as an intermediate; the crude methylene products were further oxidized when exposed to air and irradiation (conditions C, Figure 5). The authors in [80] also proposed a reaction mechanism initiated by the photoexcitation of the Ir^III^(ppy)_3_ photocatalyst followed by a single electron transfer (SET) process with the bromodifluoroacetamide reagent to afford radical **I** (Scheme 34), Br- and Ir^IV^(ppy)_3_. The electron deficient radical I (Scheme 34) undergoes a 5-*exo* cyclization to yield indole C-2 radical intermediate **II** (Scheme 34). Then, intermediate II is oxidized to carbocation **III** and the Ir^III^(ppy)_3_ is regenerated upon reduction of Ir^IV^(ppy)_3_. Then, water attacks the carbocation **III** (Scheme 34) and is followed by deprotonation, which affords the spirooxindole hemiaminal **IV** (Scheme 34) that it is finally oxidized by the addition of PCC in the same pot, generating the reaction product.

**Figure 6:**
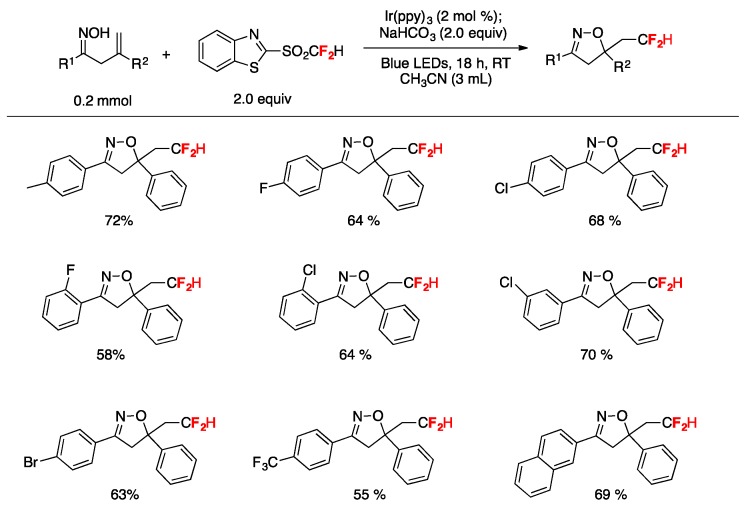
Substrate scope examples for the visible-light induced radical difluoromethylation of β,γ–unsaturated oximes.

**Scheme 35:**
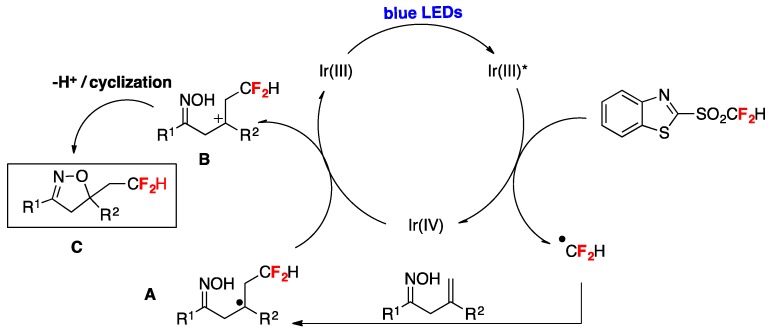
Proposed reaction mechanism for the visible-light induced radical difluoromethylation of β,γ-unsaturated oximes.

A practical method for the synthesis of difluoromethylated isoxazolines through visible-light induced radical difluoromethylation of β,γ-unsaturated oximes has been published by Zhu et al. [81]. The reaction employs an aromatic ketone-derived β,γ-unsaturated oxime as a substrate, a difluoromethylsulfone (2-((difluoromethyl)sulfonyl)benzo[*d*]thiazole) as the CF_2_H source, NaHCO_3_ as the base, and Ir(ppy)_3_ as the photocatalyst in acetonitrile as the solvent, irradiated with blue LEDs (Figure 6). Different substrates containing both electron-rich and electron-poor substituents on the phenyl ring afforded the reaction products in good yields (Figure 6). Substrates containing halogen and bulky groups (i.e., 2-naphtyl) were also well tolerated (Figure 6). Alkyl substituted oximes did not undergo the reaction under the reported conditions; the presence of an aryl substituent in conjugation with the C=C bond was found to be an important factor for the reaction to succeed due to the ability of stabilizing the CF_2_H-substituted intermediate carbocation. Regarding the mechanistic aspects, the authors in [81] proposed a domino difluoromethylation/cyclization reaction initiated upon photoexcitation of the catalyst to yield Ir(ppy)_3_*, which undergoes one electron transfer with the difluoromethylsulfone reagent affording a •CF_2_H radical and the Ir^IV^ complex. Subsequently, the •CF_2_H radical adds to the alkene of the β,γ-unsaturated oxime, affording the radical adduct **A** (Scheme 35), which reacts with the Ir^IV^ complex regenerating the Ir(ppy)_3_ catalyst and giving the CF_2_H-substituted carbocation intermediate **B** (Scheme 35). Finally, by deprotonation assisted by the base and sequential cyclization, the difluoromethylated isoxazoline product **C** is formed (Scheme 35).

**Scheme 36:**
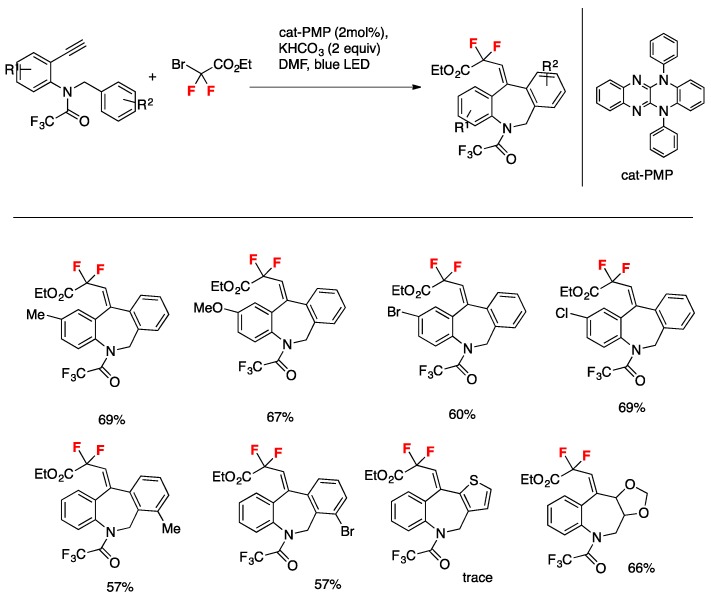
Selected examples for the synthesis of difluoro-containing dibenzazepines.

Liu, Xu, and colleagues [82] employed a novel photocatalytic system for the for efficient construction of difluoro-containing dibenzazepines. The transformation is redox-neutral and environmentally benign, has a wide range of substrate scope, proceeds at room temperature under irradiation with visible light, and furnishes dibenzazepine derivatives in mild to excellent yields. The catalyst used is 5,12-diphenyl-5,12-dihydroquinoxalino[2,3-*b*]quinoxaline, cat-PMP. The additive is KHCO_3_, in DMF as the solvent, under irradiation with a blue LED for 12 h. The scope of the transformation is illustrated in Scheme 36.

**Scheme 37:**
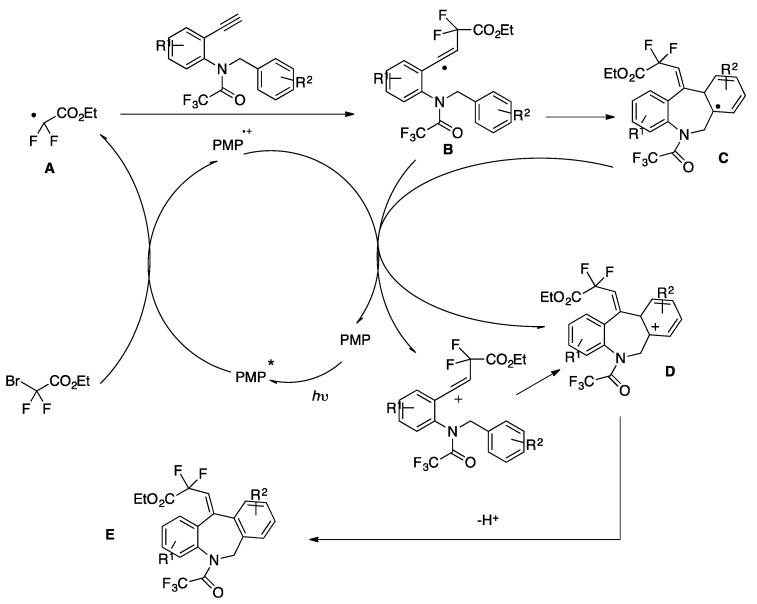
Proposed reaction mechanism.

The authors in [82] proposed a catalytic cycle (Scheme 37) that commences with the photoexcitation of cat-PMP, affording the excited-state cat-PMP*, a strong reductant (E_red_ 1/2* = −1.91 V vs. SCE) that reduces BrCF_2_CO_2_Et to its corresponding radical **A**. Radical **A** then undergoes intermolecular addition to the terminal alkynyl carbon to provide radical **B** and radical cation cat-PMP^•+^. Following the intramolecular addition of **B**, cat-PMP^•+^ accepts an electron from radical **C** and the resulting cation **D** undergoes deprotonation to furnish the desired product **E**.

**Scheme 38:**
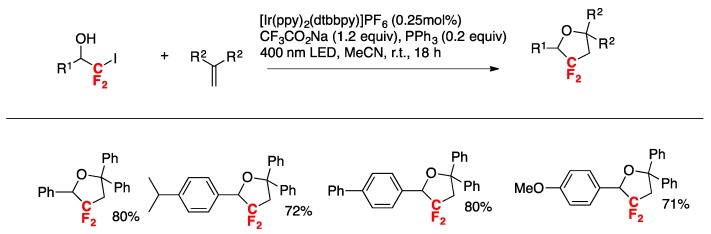
Some examples of the synthesis of 3,3-difluorotetrahydrofurans from iododifluoromethylated alcohols and 1,1-diarylethylenes.

Panferova, Dilman, and colleagues [83] accomplished the synthesis of 3,3-difluorotetrahydrofurans from iododifluoromethylated alcohols and 1,1-diarylethylenes. The scope of the transformation is illustrated in Scheme 38.

**Scheme 39:**
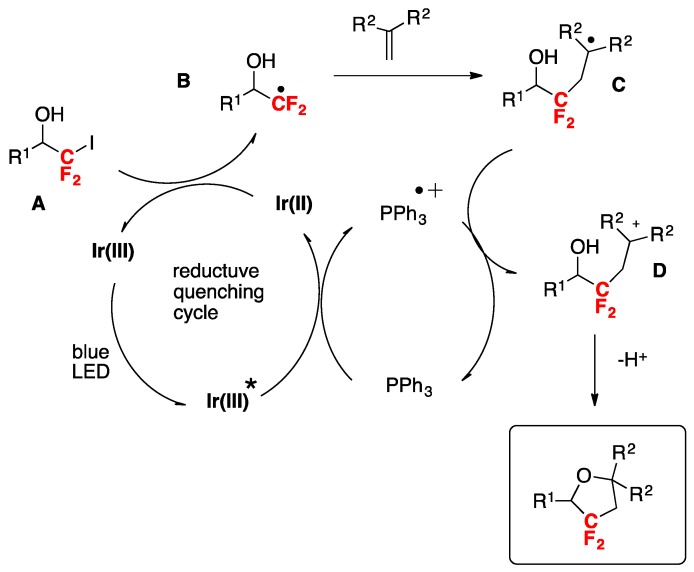
Proposed mechanism for the synthesis of 3,3-difluorotetrahydrofurans from iododifluoromethylated alcohols and 1,1-diarylethylenes.

The authors in [83] investigated the reaction mechanism and proposed the following catalytic cycles, as shown in Scheme 39.

Iodide A undergoes single electron reduction to radical B by means of the highly reductant Ir(II). Upon reaction with 1,1-diarylethene, radical **B** forms adduct C, which is oxidized to carbocation D by the radical cation of PPh_3_, which in turn is produced by ET-oxidation of excited Ir(III) and PPh_3_. Intermediate D suffers intramolecular nucleophilic attack to render 3,3-difluorotetrahydrofurans.

### 3.3. Difluoromethylation of Carbon–Carbon Multiple Bonds with Rearrangements

**Scheme 40:**
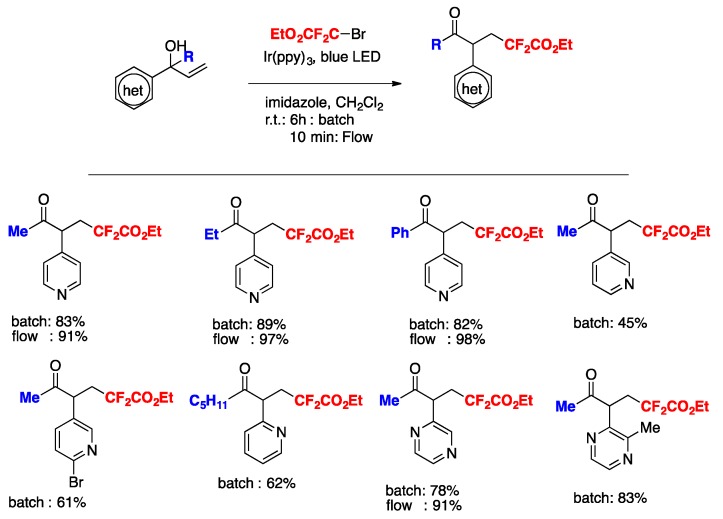
Selected examples of the Ir(III)-photocatalytic difluoromethylation reaction of heteroarenes with BrCF_2_CO_2_Et.

Wei and Noel [84] developed an Ir(III) photocatalytic difluoromethylation reaction consisting of a radical-induced heterocycle migration−variation of the allylic alcohol substrate. The scope of the transformation is represented in Scheme 40. The authors in [84] attempted both a batch reaction and a flow system. Yields reflected a higher substrate conversion under continuous flow, with a concomitant shortening of reaction times (Scheme 40).

**Scheme 41:**
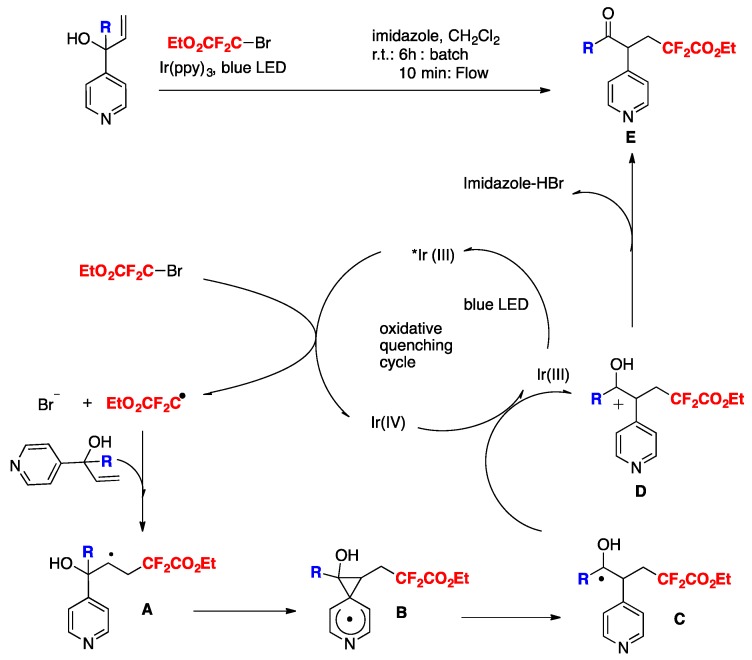
Proposed reaction mechanism.

The authors in [84] also proposed a mechanism based on the experimental data, as suggested in Scheme 41. Upon light excitation, *fac-*[Ir(ppy)_3_]* can be oxidatively quenched by BrCF_2_CO_2_Et, generating the corresponding difluoroalkyl radical species. As a matter of fact, radical trapping experiments with 2,6-di-*tert*-butyl-4-methylphenol showed that this species could be effectively trapped. The radical subsequently adds to the olefin generating intermediate **A**, which undergoes 1,2-heterocycle migration via a key spiro radical intermediate **B** to produce **C**. Finally, the intermediate **C** was oxidized (**D**) to obtain the aimed product **E** (Scheme 41).

**Scheme 42:**
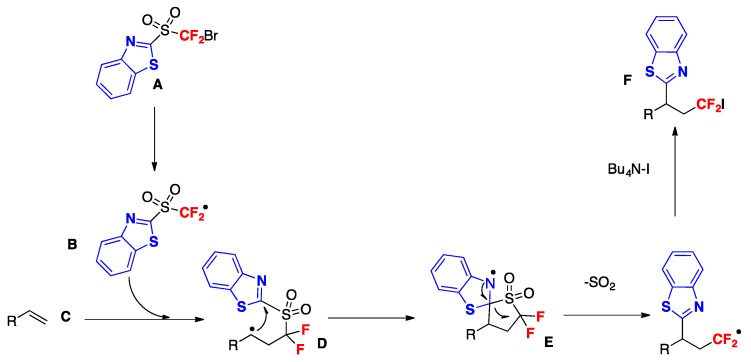
Strategy for radical-mediated difunctionalization of alkenes.

Yu, Zhu, and colleagues [85] reported a conceptually new docking–migration mode for radical difunctionalization of alkenes with the CF_2_ group. The radical donor and acceptor are contained in a dual-function reagent (**A**, Scheme 42). After docking the radical donor (**B**) onto alkene (**C**), the newly formed transient alkyl radical (**D**) attacks the radical acceptor intramolecularly (**E**), triggering the subsequent functional group migration to deliver the difunctionalized product (**F**). The conceptual basis for this transformation is illustrated in Scheme 42.

**Scheme 43:**
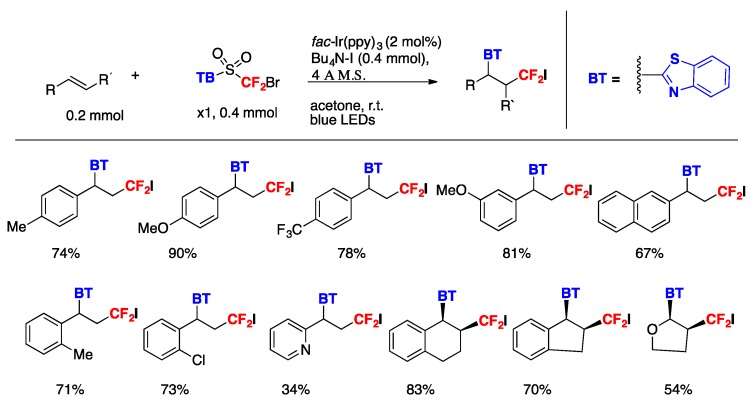
Strategies for radical-mediated difunctionalization of alkenes.

The authors in [85] generated the difluoromethyl radical from 2-((bromodifluoromethyl)sulfonyl)benzo[*d*]thiazole **A** (Scheme 42). The reaction proceeded under visible-light irradiation with *fac*-Ir(ppy)_3_ as the photocatalyst, under blue LEDs irradiation, in acetone as the solvent, at r.t. The iodine atom was derived from the additive tetrabutylammonium iodide (TBAI), which is indispensable for the conversion to furnish the desired product in good yield. The scope of the transformation is illustrated in Scheme 43.

The authors in [85] investigated the reaction mechanism conducting Stern–Volmer studies that showed that the excited fac-Ir(ppy)_3_* photocatalyst could be oxidatively quenched by A, but could not be reductively quenched by TBAI. Additionally, the reduction potential of A has a value of E_p/2_ = −0.71 V vs. SCE, indicating that the C–Br bond could easily be reduced by the Ir^III^* species (E_1/2_ Ir ^III^*^/IV^ = −1.73 V vs. SCE).

**Scheme 44:**
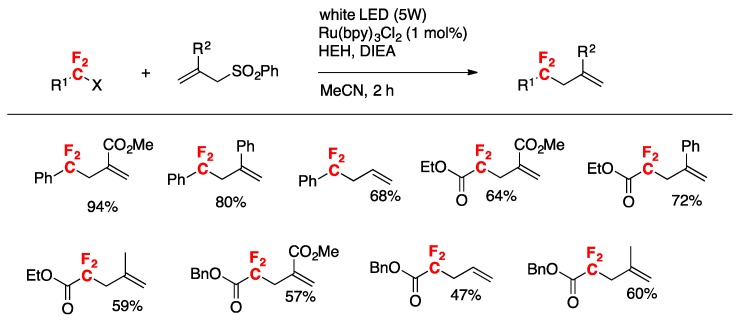
Selected examples of the photocatalytic allylation of α-fluorine-containing organic halides with allyl sulfones.

More recently, Uno, Ryu, and colleagues [86] have accomplished the photoredox-catalyzed allylation of α-gem-difluorinated organohalides with allyl sulfones, which proceeded smoothly under visible light irradiation to give 4,4-difluoroalkenes in good yields. In the presence of catalytic Ru(bpy)_3_Cl_2_, Hantzsch ester, and diisopropylethylamine, the reaction was complete within 2 h. The scope of the transformation is depicted in Scheme 44.

**Scheme 45:**
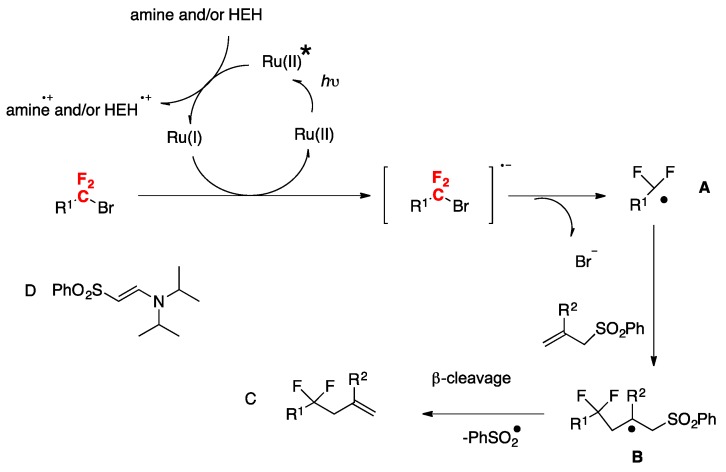
Proposed reaction mechanism.

The authors in [86] proposed the following reaction mechanism, as depicted in Scheme 45. To start with, a single electron transfer process from the Hantzsch ester (HEH) and/or amine to photoexcited Ru(II)* takes place to generate Ru(I) and the radical cation of Hantzsch ester and/or amine. Then, Ru(I) gives one electron to the difluorobromoalkyl compound to generate a difluoroalkyl radical **A** after dissociative collapse of the radical anion of the difluorobromoalkyl compound. Then, radical **A** adds to allyl sulfone to afford radical **B**, which undergoes β-fragmentation to give the allylated product **C** and phenylsulfonyl radical (PhSO_2_·). Regarding the sulfonyl radical, the one-electron reduction to PhSO_2_ anion or dimerization to disulfone ((PhSO_2_)_2_) is possible. The authors in [86] isolated other byproducts derived from the reactions such as the enamino sulfone **D**. Product **D** presumably arises from the reaction of enamine, formed via the radical cation of DIEA, with the sulfonyl radical.

**Scheme 46:**
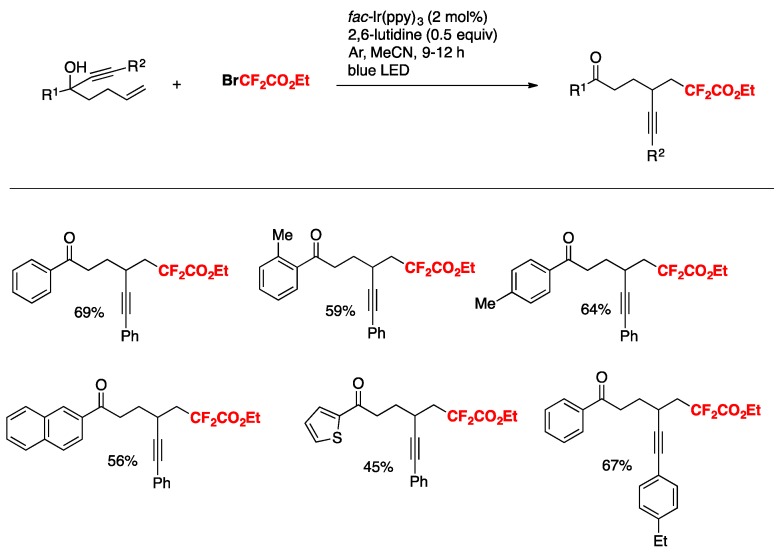
Selected examples for the visible-light-mediated tandem radical difluoroalkylation and alkynylation of unactivated alkenes.

In 2018 Liu, Xie, and Zhu [87] informed a visible-light-mediated tandem radical difluoroalkylation and alkynylation of unactivated alkenes. The protocol affords difluoroalkylated linear alkynyl ketones. The scope of the transformation is illustrated in Scheme 46.

**Scheme 47:**
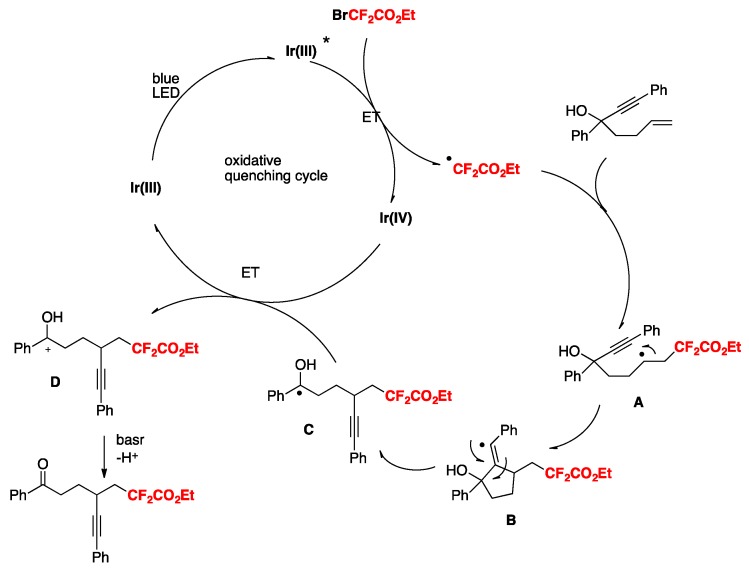
Proposed reaction mechanism.

The authors in [87] explored the reaction mechanism, and proposed a catalytic cycle such as that shown in Scheme 47. The strongly-reducing excited Ir(III) photocatalyst is quenched by BrCF**_2_**CO**_2_**Et to produce CF**_2_**CO**_2_**Et radicals (generating Ir(IV) in the process). The CF_2_CO_2_Et radical adds to the terminal olefinic carbon affording radical adduct A. Radical adduct A undergoes intramolecular 5-exo-cyclization to the alkynyl carbon to produce the vinyl radical B. Subsequent homolysis of the Csp**^3^**–Csp**^2^** bond affords the hydroxyalkyl radical C, which by Ir(IV)-induced oxidation gives carbocation D and the rearranged product [88].

## 4. Conclusions

A wide range of synthetic photocatalytic methodologies have been presented to accomplish the introduction of CF_2_H/CF_2_R groups into unsaturated systems. However, few of the photocatalytic strategies presented involve *all-organic* photocatalysts. Further work is needed in the areas of photocatalysis, especially in transition metal-free strategies involving organic dyes and other metal-free organic compounds aimed at producing the CF_2_ radical species. This is of particular concern in the pharmaceutical industry to avoid the use of transition metals. Another area that deserves particular attention, in terms of difluoromethylation strategies, is the application of flow systems to achieve CF_2_H substitutions in high yields and minimal reaction times. This area, that is, flow systems, has received particular attention for trifluoromethylation, perfluoroalkylation, and fluorination reactions, however, few reports of a difluoromethylation flow system have been documented.
